# Sustainable Synaptic Device with Two‐Dimensional Ferroelectric Materials for Neuromorphic Computing

**DOI:** 10.1002/advs.202600064

**Published:** 2026-05-07

**Authors:** Jaewook Yoo, Seokjin Oh, Minah Park, Jong Min Song, Hongseung Lee, Seohyeon Park, Sojin Jung, Seongbin Lim, Soohyun Lim, Dongsun Shin, Ji Won Heo, TaeWan Kim, Hagyoul Bae

**Affiliations:** ^1^ Division of Electronic Engineering Jeonbuk National University Jeonju Republic of Korea; ^2^ HB Inc. Jeonju Republic of Korea; ^3^ School of Advanced Fusion Studies and AI Semiconductor University of Seoul Seoul Republic of Korea; ^4^ 2D Epi, Inc. Jeonju Republic of Korea

**Keywords:** 2D ferroelectrics, convolution neural networks (CNN), current annealing (CA), sustainable synaptic devices, α‐In_2_Se_3_

## Abstract

To overcome the coming big‐data era, significant efforts are needed to implement neuromorphic computing that mimics the functional and structural features of the human brain using electronic devices. Repeated updates mimicking biological synaptic plasticity degrade the endurance of synaptic devices during in situ training. This study demonstrates a two‐dimensional (2D) ferroelectric α‐In_2_Se_3_‐based synaptic device with enhanced durability via self‐curing performed from current annealing (CA) in synaptic fatigue occurring during repetitive learning. The conduction, degradation, and self‐curing mechanisms of the 2D ferroelectric‐based synaptic device are quantitatively elucidated by low‐frequency noise (LFN) spectroscopy. The classification accuracy of the Canadian Institute for Advanced Research (CIFAR)‐10 dataset with self‐cured conductance is superior to that of the device with synaptic fatigue and recovers to the initial accuracy level. The simulation results of removing defect cells through self‐curing in the 2D ferroelectric synaptic array can help reduce energy consumption in the long term. The experimental results emphasize adopting 2D ferroelectric materials for future neuromorphic computing.

## Graphical abstract

α‐In_2_Se_3_ based FeSFETs can be utilized as sustainable devices through polarization switching governed by both out‐of‐plane and in‐plane polarizations. Upon reaching a fatigued state, current annealing enabled by conductance modulation can significantly enhance the endurance of FeSFETs. Moreover, current annealing can be selectively and dynamically applied to defective cells in real time at the system level, while maintaining a desirable and optimized power consumption.

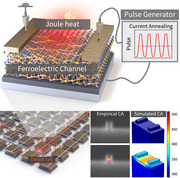

## 1 Introduction

Remarkable advances in image processing performance, particularly in recognition, segmentation, and object detection, have been driven by convolutional neural networks (CNNs) that integrate multiple convolutional and subsampling (pooling) layers [1, 2]. Owing to their shared weights and localized connections, CNNs can effectively extract salient feature information without the need for extensive input data engineering [3, 4, 5]. However, when deployed on conventional von Neumann architectures, CNNs inevitably suffer from increased power consumption, heat dissipation, and limited computational speed, primarily due to massive data transfer between the processing and memory units [6, 7]. To overcome these limitations, neuromorphic computing systems integrate processing and memory functions by mimicking the biological brain, thereby breaking away from the von Neumann bottleneck. For neuromorphic computing to emerge as a compelling computational paradigm, two key objectives must be achieved: (1) massive parallelism, in which a single neuron connects to thousands of others through synapses, and (2) adaptability and resilience against distorted or faulty input signals [8, 9]. Nevertheless, large‐scale parallel processing in biological synapses can lead to synaptic fatigue, consequently resulting in the transmission of ambiguous or unreliable information [10, 11]. To address this, biological systems employ recovery mechanisms that restore synaptic efficacy, thereby preventing potential functional failure or disorder [12].

Ferroelectric field‐effect transistors (FeFETs) have attracted significant attention as artificial synaptic devices owing to their high power efficiency and fast switching characteristics, which enable the emulation of synaptic plasticity. Such devices have been developed by integrating ferroelectric materials, such as HfO_2_‐based compounds, perovskites, and P(VDF‐TrFE), into the gate stack [13, 14, 15, 16, 17]. However, conventional ferroelectric materials require a minimum critical thickness to maintain stable ferroelectricity, and additional dielectric layers are often introduced to suppress leakage current [18]. Moreover, when the ferroelectric layer is scaled down to the nanometer regime, the inevitable formation of a low‐permittivity dead layer makes the device highly susceptible to depolarization fields, thereby limiting its long‐term reliability [13, 19].

Among two‐dimensional (2D) materials, material α‐In_2_Se_3_ can be directly utilized as a semiconducting channel exhibiting intrinsic ferroelectricity due to its asymmetric atomic structure, thus overcoming the reliability degradation issues inherent in conventional gate stacks [20, 21]. Furthermore, 2D materials possess exceptional physical properties, including atomically thin layered structures, dangling‐bond‐free surfaces, high electrical robustness, and superior carrier mobility, making them ideal candidates for high‐performance synaptic devices. α‐In_2_Se_3_, which exhibits in‐plane (IP) or out‐of‐plane (OOP) ferroelectricity at room temperature, also demonstrates outstanding optoelectronic and thermoelectric properties, enabling its application in a wide range of fields, such as nonvolatile memory, neuromorphic and in‐memory computing, optoelectronic devices, photodetectors, and photovoltaic systems [22, 23, 24, 25, 26, 27, 28]. Nevertheless, research on α‐In_2_Se_3_‐based ferroelectric semiconductor field‐effect transistors (FeSFETs) has predominantly focused on their functional applications, largely due to the scarcity of intrinsic ferroelectric channel materials. Efforts aimed at improving performance and mitigating degradation have also remained primarily material‐oriented, with most studies relying on simulation‐based analyses rather than experimental validation.

In this context, low‐frequency noise (LFN) spectroscopy can serve as an insightful analytical technique for elucidating the electrical conduction mechanism of FeSFETs. Conventional approaches used for analyzing α‐In_2_Se_3,_ such as probe‐based methods including piezoresponse force microscopy (PFM) and Kelvin probe force microscopy (KPFM), as well as optical techniques like X‐ray photoelectron spectroscopy (XPS) and Raman spectroscopy, primarily probe the system in a static state, and therefore fail to fully capture the dynamic behavior relevant to actual device performance [22, 23, 24]. In contrast, LFN, which is obtained through Fourier transformation of the time‐domain signal, enables real‐time analysis of charge‐trapping and detrapping events at the gate oxide/channel interface, as well as carrier scattering dynamics within the channel [29, 30, 31]. Furthermore, because LFN determines the ultimate signal floor of analog and digital integrated circuits based on nanoscale electronic materials and devices, it represents a critical metric that must be thoroughly investigated prior to the development of large‐scale integrated arrays in the future [32].

Through this study, we demonstrate that α‐In_2_Se_3_‐based FeSFET can function as sustainable synaptic devices. Previous studies have predominantly focused on improving the synaptic transmission process; however, research addressing the synaptic reuptake mechanism, which counteracts inevitable synaptic depression in both biological and artificial systems, has been largely absent. The sustainable synaptic device was implemented using a self‐curing current annealing (CA) process approach, in which high‐current‐induced Joule heat is momentarily induced by controlling electrical conductivity (*σ*). This process offers strong practical potential, as it enables localized curing at the chip level without requiring external equipment or integrated modules, particularly when combined with recently reported large‐area–grown thin films [33]. Moreover, earlier FeSFET research has primarily concentrated on nonvolatile memory applications and endurance based on digital resistive switching. In this work, however, both the electrical conduction and degradation mechanisms are comprehensively analyzed through LFN spectroscopy, providing critical insights that can serve as guidelines for the broader utilization of FeSFET technology. By coupling this technological approach with dynamic electrical analysis, our results establish a foundational framework for synaptic devices capable of maintaining accurate data processing even under large‐scale parallel operation, paving the way toward neuromorphic architectures with sustainable functionality.

## 2 Results and Discussion

**FIGURE 1:**
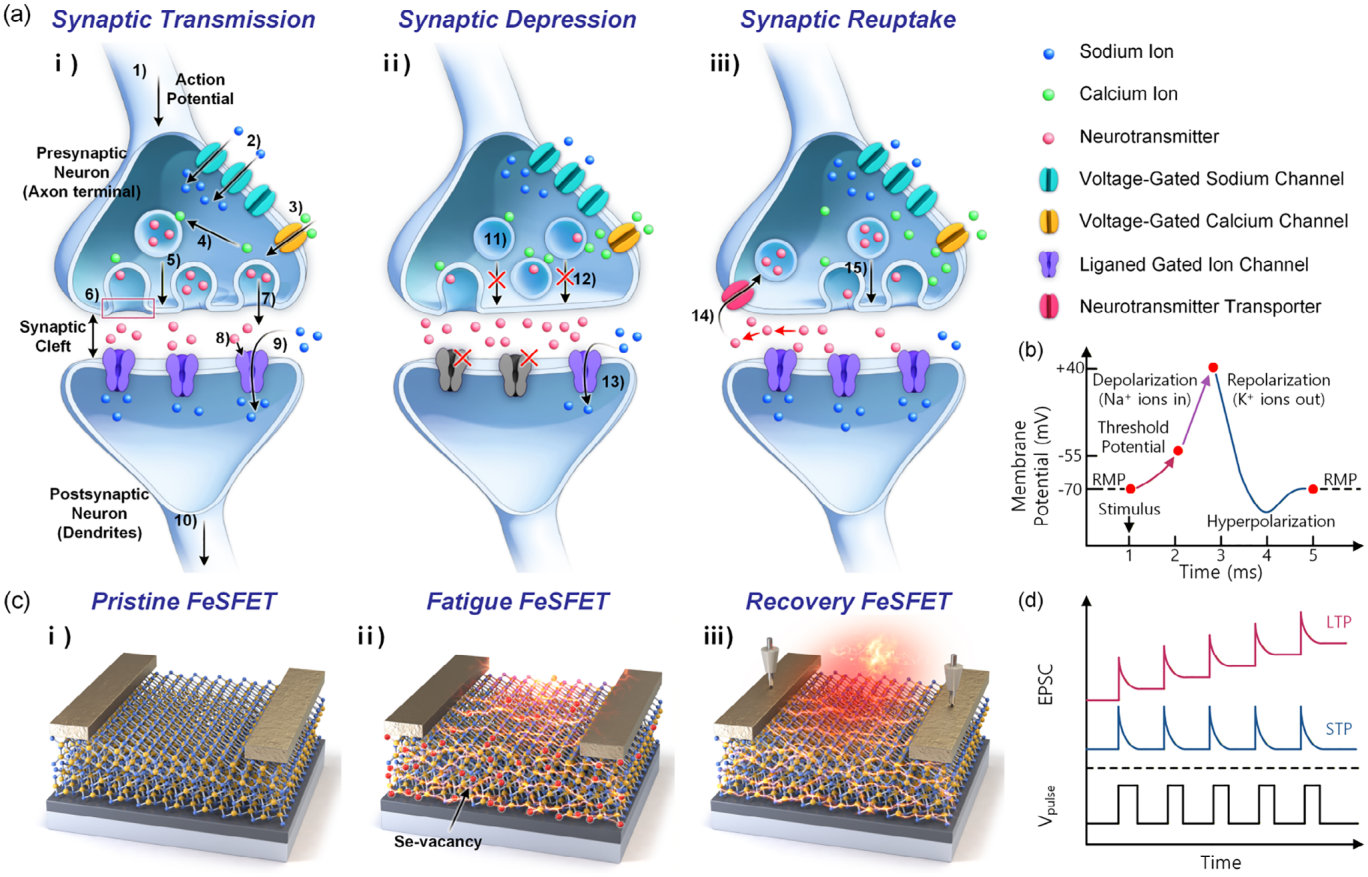
(a) Stepwise schematic of biological synaptic activity, showing (i) synaptic transmission, (ii) synaptic depression under repetitive stimulation, and (iii) recovery via reuptake and vesicle refilling. (b) Variation of membrane potential during the synaptic transmission process. (c) Stepwise schematic of artificial synapse, showing (i) pristine, (ii) fatigue, and (iii) recovery state in α‐In_2_Se_3_‐based FeSFET. (d) LTP and STP of the artificial synapse.

The synaptic transmission process that occurs when an external stimulus is applied to a biological neuron is illustrated in Figure 1a [34]. Each step of this process is sequentially numbered for clarity. An action potential refers to a rapid and transient change in membrane potential that occurs in certain excitable cells, such as neurons and muscle cells. Under resting conditions, the presynaptic neuron maintains a resting membrane potential (RMP) of approximately −70 mV, as potassium channels remain partially open [35]. When sodium ions enter the cell, and the membrane potential reaches the threshold level, depolarization occurs, leading to the opening of calcium channels and the subsequent influx of calcium ions. The synaptotagmin proteins located in synaptic vesicles then bind to the incoming calcium ions, triggering exocytosis of neurotransmitters into the synaptic cleft [36]. Simultaneously, potassium efflux induces repolarization, reducing the action potential amplitude and leading to hyperpolarization, after which the neuron gradually returns to its resting potential. This sequence is schematically shown in Figure 1b. The released neurotransmitters bind to ionotropic receptor channels on the postsynaptic neuron, allowing sodium ions to flow inward and thereby transmitting the stimulus from the presynaptic side [10, 11]. Figure 1a‐ii,iii further illustrate the synaptic reuptake process, which compensates for synaptic depression. Synaptic depression arises when neurons are stimulated with high frequency or intensity, resulting in excessive accumulation of residual neurotransmitters in the synaptic cleft and depletion of vesicles in the presynaptic terminal, ultimately distorting synaptic conductivity [12, 37]. To counter this, the synaptic reuptake mechanism in the axon terminal retrieves neurotransmitters from the synaptic cleft back into the cytoplasm, refilling the emptied vesicles and restoring normal synaptic transmission. The processes illustrated in Figure 1a correspond sequentially to the (i) pristine, (ii) fatigue, and (iii) recovery states of the α‐In_2_Se_3_‐based FeSFET, as depicted in Figure 1c. Figure 1d illustrates how variations in conductance are mapped onto synaptic weights through short‐term plasticity (STP) and long‐term plasticity (LTP), thereby mimicking the plastic behavior of biological synapses in terms of memory retention using an artificial synapse. The fatigue state shown in Figure 1c introduces critical errors in conductance modulation required for short‐ and long‐term memory retention, which severely hinders the successful emulation of biological synaptic behavior in FeSFETs.

This biological process closely parallels the degradation and recovery phenomena observed in transistor devices. Continuous transistor operation induces electrical and thermal stress in the gate dielectric and channel layers, analogous to neuronal fatigue in biological systems. Thus, the effort to utilize transistors as synaptic devices by modulating channel conductivity rather than simply storing binary states (0 and 1) in the gate oxide must address this degradation issue. Historically, CA techniques have been employed in silicon‐based dielectric layers, oxide semiconductors, hafnium‐based ferroelectric materials, and two‐dimensional materials to recover device performance by generating localized high temperatures through transient high‐current flow [38, 39, 40, 41, 42]. This recovery mechanism can be regarded as the electronic analogue of the synaptic reuptake and vesicle refilling process in biological neural systems.

**FIGURE 2:**
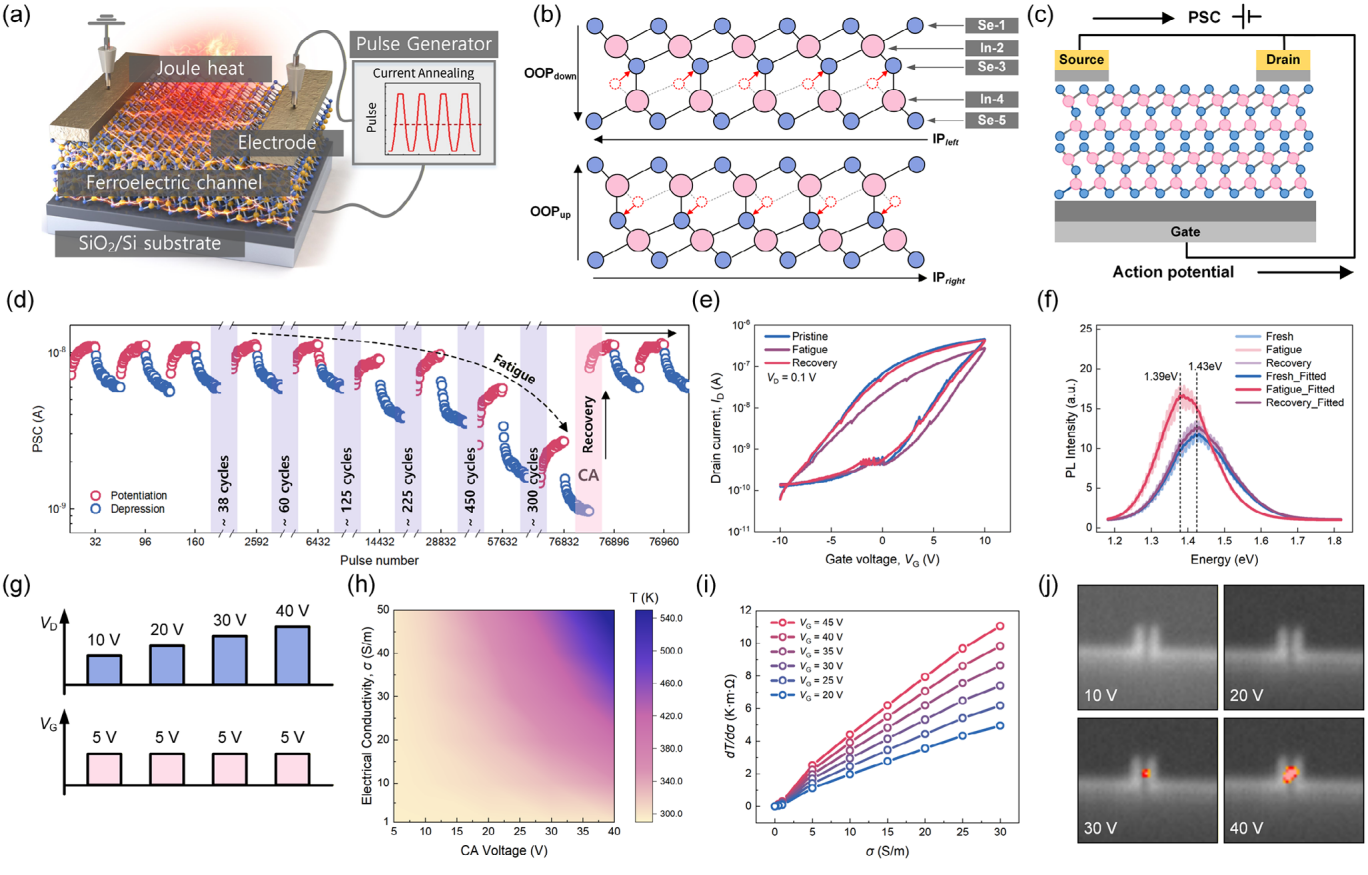
(a) 3D schematic of the vdW α‐In_2_Se_3_ FeSFET and Joule heat generated during the CA process. (b) Atomic configuration of monolayer α‐In_2_Se_3_ composed of In and Se atoms, showing the crystal lattice and direction of OOP and IP polarizations. (c) Schematic of the FeSFET operation in which a gate stimulus induces a PSC flowing from the source to the drain. (d) 5‐bit potentiation/depression behavior of the FeSFET showing PSC degradation after 1200 cycles and recovery to the initial state after a single CA process. (e) Transfer characteristics of the FeSFET measured in the pristine, fatigue, and recovery conditions. (f) Room‐temperature PL spectra of the same device under identical conditions for each state. (g) Summary of the voltage conditions used for the CA process. (h) Simulated temperature distribution in the α‐In_2_Se_3_ channel as a function of applied CA voltage and electrical conductivity. (i) Differentiated temperature over conductivity according to electrical conductivity (*σ*). (j) Voltage‐dependent thermal emission images obtained using the THEMOS system.

Figure 2a shows the device configuration of the α‐In_2_Se_3_‐FeSFET and the positive drain pulses applied for CA process. The fabricated FeSFET exhibits synaptic fatigue due to the degradation of LTP/LTD characteristics caused by repetitive weight updates. The discussion and clarification of this mechanism will be presented in a later section. Figure 2b illustrates the out‐of‐plane (OOP) and in‐plane (IP) polarizations that originate from ferroelectricity, coupled respectively with the vertical (gate) and horizontal (drain‐to‐source) electric fields [26]. These polarizations simultaneously align the dipoles, modulating the energy band and resulting in two resistance states: low‐resistance state (LRS) and high‐resistance state (HRS), which induce a clockwise hysteresis loop. Figure S1a presents the optical microscope image of the fabricated FeSFET with a channel length of 4 µm. The thickness measured by atomic force microscope (AFM) in Figure S1b is approximately 15 nm, corresponding to 11–13 layers of α‐In_2_Se_3_ flake. The FeSFETs used in this study were fabricated using α‐In_2_Se_3_ flakes with *t*_ch_ ranging from 15 to 30 nm. The seven Raman spectra shown in Figure S1c support the bulk‐layer thickness information of the α‐In_2_Se_3_ flake used for FeSFET fabrication. In this study, as shown in Figure 2c, FeSFET was used to emulate LTP. As shown in Figure 2d, the FeSFET exhibits a 5‐bit potentiation/depression behavior, where the conductance decreases by approximately 50% after 1,200 repeated cycles. This reduction in conductance may originate from accumulated defects in the gate oxide or within the α‐In_2_Se_3_ bulk layer. This phenomenon is consistent with synaptic depression observed in biological neurons. Additional data on the potentiation/depression cycling and the sustainability of the CA process are provided in Figure S2. The programming voltage for extracting the LTP characteristics was optimized to reflect symmetric current levels and low nonlinearity (NL) in the system‐level simulations. The device performance remains stable for at least 4,000 cycles (256k pulses). Figure 2e shows the transfer characteristics of the FeSFET measured in three states: pristine, fatigue (after 4000 cycles), and recovery (after CA). Compared with the pristine state, the LRS in the fatigue condition exhibits significant degradation, but it is almost completely restored after the CA process. After 4000 cycles, the effect of the subsequently applied CA process was preserved without degradation even after four months. The LTP/LTD characteristics measured over 1000 cycles under this condition are presented in Figure S3a. The fatigue state is induced by repeated cycling in the FeSFET, and the effect of the re‐applied CA process exhibits consistent behavior. As discussed earlier, the LTP/LTD measurements employing asymmetric pulse amplitudes to achieve symmetric current levels and low nonlinearity may introduce the imprint effect. In addition, to enable the FeSFET to represent a high number of conductance states, a sufficiently wide dynamic range must be secured. To address this issue, LTP/LTD pulses with identical amplitudes but higher magnitudes than the previous conditions were applied. Figure S3b shows the results obtained consecutively after Figure S3a, where the LTP/LTD characteristics over 500 cycles were measured using the pulse conditions shown in Figure S3c. Similar to the previous case, the device exhibited a fatigue state, after which the optimized CA process was applied. With an induced current of approximately 18 µA, the FeSFET was recovered to a state close to its initial condition. Although the device degradation was more pronounced than in the previous condition, resulting in a relatively lower induced current, the successful execution of the CA process remains encouraging. Figure S3d presents the transfer characteristics measured under each condition discussed in Figure S3. The observed fatigue state and CA‐induced recovery effects originate from trap related reactions at the interface and within the gate oxide, which will be discussed in detail in a later section.

Figure 2f shows the room‐temperature PL spectra of α‐In_2_Se_3_ corresponding to the three states. After fatigue, the PL peak exhibits a red shift of approximately 0.04 eV, accompanied by a 140% increase in intensity. This indicates that the increased defect density in the thin film promotes recombination with lower energy emission [43]. After the CA process, the PL peak blue‐shifts to its initial position, and the intensity recovers to about 107% of the pristine level. The Raman spectra shown in Figure S4 exhibit no discernible change after the operation. This observation is consistent with previous reports that sulfur vacancies in MoS_2_ form mid‐gap states within the bandgap, where long‐term electrical stress can induce redistribution of sulfur vacancies in the channel while causing only negligible lattice damage [44]. Similarly, Se vacancies have been simulated to form at specific energy levels, and no significant lattice distortion is observed in the Raman spectra [45]. To investigate the origin of the fatigue state, X‐ray photoelectron spectroscopy (XPS) spectra were introduced, as shown in Figure S5. The In and Se core‐level peaks shift toward lower binding energies by approximately 0.3 eV and 0.6 eV, respectively, in the fatigue state. This shift indicates that electrons previously involved in In─Se bonding are converted into free electrons due to the formation of Se vacancies, which simultaneously induces enhanced electronic screening [46]. After CA, the In and Se peaks recover to positions similar to those of the pristine state, suggesting that Joule heating during CA can effectively reduce Se vacancies. Meanwhile, an increase in defect density can affect the Schottky barrier height (Φ_B_) at the metal–semiconductor junction, contributing to increased contact resistance and consequent degradation of device performance. The Φ_B_ can be extracted from thermionic emission current, and to examine this effect, Arrhenius plots (Figure S6a–c) and the corresponding Φ_B_ (Figure S6d–f) were evaluated for each device state, as shown in Figure S6 [32]. As observed in Figure S6a–c, when the drain voltage transitions from negative to positive, the polarization switches to the opposite direction; accordingly, the $\ln\;(I_{D}/T^{3/2})$ terms decrease under negative drain bias and increase under positive drain bias. Because the polarization‐switching‐induced potential has an opposite polarity to the applied electric field, it can effectively induce a lower Φ_B_ than the theoretical value [47]. Figure S6d–f shows that, under identical drain‐voltage conditions, the Φ_B_ in the fatigue state increases compared to that in the pristine state, whereas it decreases again in the recovery state after the CA process. Notably, the extent of Φ_B_ modulation with drain voltage is significantly more pronounced in the fatigue state than in the pristine and recovery states, under both negative and positive bias conditions. Figure S6g illustrates the corresponding energy‐band diagram. Defects generated at the junction interface and within the α‐In_2_Se_3_ bulk in the fatigue state enhance charge trapping and de‐trapping processes, which in turn screen the intrinsic IP polarization dipoles of α‐In_2_Se_3_ and suppress Schottky barrier lowering. After applying the CA process to the fatigued device, the defect density is recovered toward its original level, allowing both the polarization and the Φ_B_ to be restored.

As shown in Figure 2g, the CA process was performed by first applying a 5 V gate bias to accumulate electrons, followed by a drain pulse of *V*_pulse_ = 40 V with a pulse width of *t*_pulse_ = 1 s. During this process, a current of approximately 20 µA generated Joule heat, as confirmed in Figure S7a. Figure S7b presents the output characteristics under positive and negative poling conditions, where the resistance change increased by approximately twofold. This effect becomes more pronounced with stronger OOP and IP polarization switching. Therefore, the OOP polarization switching induced by the gate field can effectively modulate the electrical conductivity of the α‐In_2_Se_3_. Experiments designed to assess the relative contributions of current and temperature in the CA process are presented in Figure S8. Figure S8a,b shows the output characteristics of the FeSFET under different gate voltages, and the corresponding transfer characteristics after recovery state using different CA process cases, respectively. Although identical current levels (20 µA) were achieved by independently tuning the gate and drain voltages, the CA effect was negligible when the gate voltage exceeded the drain voltage, compared to the case where a higher drain voltage was applied. This result indicates that attaining a specific current level alone does not guarantee successful CA; rather, emphasis should be placed on engineering the Joule heating induced by carriers injected under the drain–source electric field. Furthermore, considering that the back‐gated FeSFET structure investigated here has a significantly larger physical scale in the drain–source direction than in the gate–channel direction, continued channel‐length scaling is expected to enable the use of lower drain voltages, offering advantages in terms of power efficiency.

The temperature variation within the channel caused by the CA pulse was estimated using finite‐element simulations based on COMSOL Multiphysics. Additional simulation results and the parameters used are summarized in Figure S9 and Table S1. Figure S9a presents the side‐view heat distribution profile, clearly showing that the highest temperature is concentrated at the center of the channel and near the interface region. Furthermore, Figure S9b,c reveals a broad distribution of Joule heat depending on the *t*_ch_ and channel length (*L*_ch_), highlighting the need for optimization according to the structural parameters of the FeSFET. Figure S10 presents the CA efficiency as a function of *t*_ch_ using FeSFETs with different thicknesses. Figure S10a,b shows the transfer characteristics of FeSFETs with *t*_ch_ of 11 nm and 70 nm, respectively. In back‐gated FeSFETs, thicker channels experience limited polarization switching under the effective electric field, while the increased charge supply within the channel leads to difficulties in off‐current control due to enhanced drain–source leakage current. Figure S10c summarizes the fatigue efficiency (η_Fatigue_), recovery efficiency (η_Recovery_), and CA efficiency (η_CA_) calculated based on the current extracted from the measured transfer curves at *V_G_* =  10 V and *V_D_* =  0.1 V, with the corresponding equations included in the Figure S10c. To ensure an equivalent comparison across different *t*_ch_, the pristine, fatigue, and recovery states were evaluated under the same experimental conditions as those used in the previous experiment (Figure 2e). As the *t*_ch_ decreases, effective polarization switching occurs, and the electric field becomes increasingly concentrated, resulting in a sharp increase in fatigue state. This implies that degradation is relatively suppressed as the distance from the interface increases. After the CA process, thinner‐channel devices exhibit approximately 4% degradation relative to the initial on‐current. Note that recovery efficiency approaches the ideal condition as it approaches 0%. From the CA efficiency analysis, it is evident that although thinner channels show reduced tolerance to pulse endurance, the necessity of the CA process becomes more pronounced.

Figure 2h presents the simulated temperature distribution as a function of *V*_pulse_ and *σ*. Because *σ* is closely related to the degree of polarization switching, it must be carefully considered when evaluating Joule heating effects. Both the annealing temperature and the amount of heat generated increase with higher *σ* and applied voltage, suggesting the possibility of unintended thermal intensity under strong polarization switching conditions. It should be noted that previous studies have reported a phase transition from the α‐phase to the β‐phase in In_2_Se_3_ when subjected to post‐electrical annealing (PEA) temperatures above 423 K [47]. Figure 2i shows the gradient of channel temperature as a function of *σ*. At *σ* of 30 S/m and an applied CA voltage of 45 V, the channel temperature changes by approximately 11 K for every 1 S/m variation in conductivity. Therefore, the CA process must be carefully optimized, as applying excessively high voltages or inducing excessive polarization switching can result in either abrupt or insufficient annealing temperatures, potentially leading to nonuniform device recovery and undesired phase transitions. To anticipate FeSFET device miniaturization, CA simulations were further performed by varying the *t*_ch_ and *L*_ch_, as shown in Figure S9d,e. The simulations reveal that, under the same CA pulse conditions, the temperature distribution within the channel depends strongly on the device geometry, highlighting the need for scale‐dependent CA pulse design to ensure uniform thermal effects across different dimensions. Figure 2j displays the images captured by a thermal emission microscope (THEMOS), showing that the generated heat increases proportionally with the applied voltage. The simultaneously captured optical emission signals are provided in Figure S11.

**FIGURE 3:**
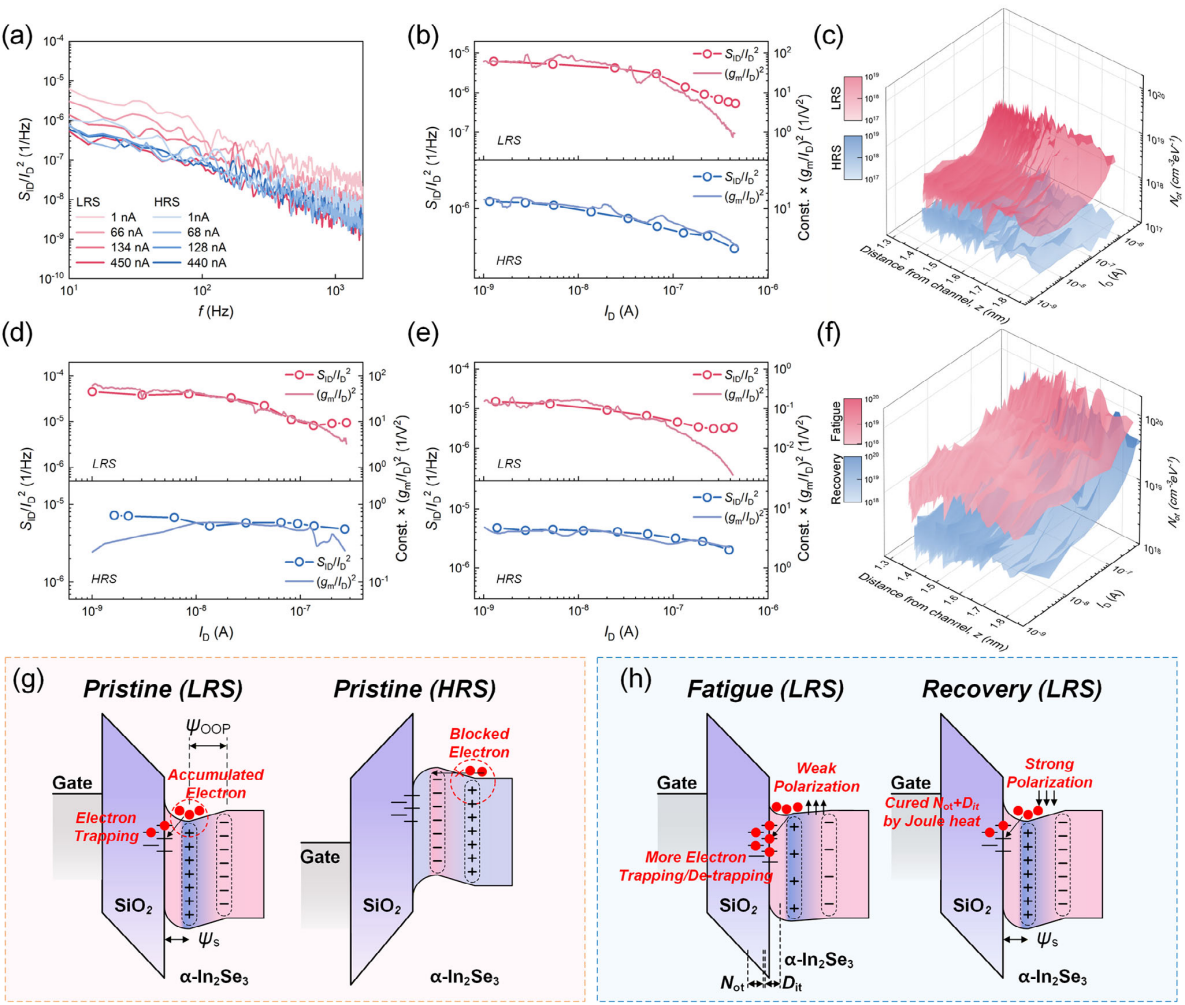
Noise and defect analysis of the α‐In_2_Se_3_ FeSFET in both LRS and HRS: (a) *S*_ID_/*I*_D_^2^ vs. frequency. (b) *S*_ID_/*I*_D_^2^ values sampled at 10 Hz and (*g*_m_/*I*_D_)^2^ values vs. *I*_D_. (c) 3D distribution of the *N*_ot_ obtained from PSD. *S*_ID_/*I*_D_^2^ values sampled at 10 Hz and (*g*_m_/*I*_D_)^2^ values vs. *I*_D_ (d) fatigue state, (e) recovery state. (f) 3D distribution of the *N*_ot_ obtained from PSD compared to fatigue and recovery state. Conceptual illustration of (g) surface potential of the FeSFET in the pristine state, depending on the resistance state (polarization direction), (h) the increased *N*_ot_ in the gate oxide and weakened polarization in the fatigue state, and their recovery after the CA process, showing reduced traps and enhanced polarization.

To quantitatively elucidate the fatigue phenomenon observed in the FeSFET under repeated weight updates (as shown in Figure 2d), we introduced LFN spectroscopy. As a noise‐based analytical approach, LFN spectroscopy offers deep insight into the electrical reliability and conduction dynamics of ferroelectric systems, allowing simultaneous assessment of interfacial and bulk defect states [39, 48]. The measurement setup used to extract the noise characteristics of the FeSFET is described in Figure S12. We first analyze the electrical conduction mechanisms of the FeSFET according to its polarization direction using the LFN technique. Figure 3a presents the normalized drain current noise power spectral density (PSD, *S*_ID_/*I*_D_^2^) as a function of frequency (*f*) for both the low‐resistance state LRS and HRS of the pristine FeSFET, measured under identical sampling currents. The unnormalized PSD data (*S*_ID_) are provided in Figure S13. Under all bias conditions, the FeSFET exhibits a 1/*f* noise behavior, indicating a flicker‐noise‐dominated conduction process. To further explore the physical origin of this 1/*f* noise behavior, Figure 3b plots the normalized noise PSD as a function of *I*_D_, demonstrating that both resistance states follow the characteristics of the carrier number fluctuation (CNF) model, proportional to (*g*_m_/*I*_D_)^2^ [30]. The CNF model attributes 1/*f* noise to the random trapping/de‐trapping of carriers within the gate oxide trap, which can also induce fluctuations in the flat‐band voltage (*V*_fb_), and can be expressed as follows:

(1) $$\frac{S_{ID}}{I_{D}^{2}}={\left(\frac{g_{m}}{I_{D}}\right)}^{2}\;S_{Vfb}$$

where *g*_m_ is transconductance, *S*_Vfb_ is the *V*_fb_ fluctuation. The *S*_Vfb_ is expressed as

(2) $$S_{Vfb}=\frac{q^{2}kTN_{ot}\lambda }{WLC_{ox}^{2}f}\;$$

where *q* is the electron charge, *k* is the Boltzmann constant, *T* is the temperature, *N*_ot_ is the volume of oxide trap density, λ is the tunneling attenuation coefficient, *WL* is the effective area of the gate, and *C*_ox_ is the gate oxide capacitance per unit area, and *f* is the frequency. Figure 3b shows that the FeSFET exhibits a higher noise level in the LRS compared with the HRS within the measured current range. In particular, excess noise is observed at drain currents exceeding 100 nA. This behavior is primarily attributed to contact‐resistance‐induced noise, which becomes more pronounced when the device is in the LRS [49, 50]. Because the channel resistance decreases in the LRS, the contribution of contact resistance is magnified in the series resistance model that includes both contact and channel resistances [32]. A previous study on MoS_2_ FETs has reported that excess noise arising from sulfur vacancies appears at low *I*_D_ levels but can be eliminated through thermal annealing [49]. Therefore, by correlating the PL spectroscopy and XPS results with the LFN analysis, the CA process is inferred to be an effective method for suppressing Se vacancies in α‐In_2_Se_3_‐based FeSFETs [51].

The frequency domain of the PSD can be converted into the corresponding vertical distance from the interface to the gate oxide using the following relation:

(3) $$z=\lambda \ln\left(\frac{1}{2\pi f\tau_{0}}\right)\;$$

where τ_0_ is the time for tunneling into a trap state at the interface (*z*  = 0). Figure 3c profiles the *N*_ot_ of the FeSFET as a function of its polarization state. As the OOP polarization switching becomes more pronounced (corresponding to higher *I*_D_), the degree of charge trapping/de‐trapping in the gate‐oxide increases accordingly. Figure 3d,e demonstrates that both the fatigue and recovery devices follow the CNF model, while the noise level in the fatigue state is clearly higher than that in the pristine or recovery states. Moreover, in the fatigue state, excess noise is observed below 10 nA in the HRS, which disappears after recovery. Figure 3f presents the extracted *N*_ot_ for the fatigue and recovery states. In the fatigue condition, *N*_ot_ increases overall across the measured current range, with particularly strong enhancement under weak gate fields due to excess noise. Ideally, the *N*_ot_ within the SiO_2_ layer should remain constant regardless of polarization direction; however, Figure 3c reveals that it varies significantly by approximately sixfold at 1 nA and over twentyfold at 1 µA. This behavior can be explained by considering the combined effects of (1) polarization switching and (2) gate stack potential, as illustrated in the energy band diagrams in Figure 3g. When a positive or negative gate voltage is applied to establish the HRS and LRS, respectively, the OOP potential (*Ψ*_OOP_) induced near the interface assumes the opposite polarity of the gate voltage. In the LRS, the *Ψ*_OOP_ formed by positive polarization near the interface facilitates electron accumulation at the interface, thereby enhancing trapping in the gate oxide. In contrast, in the HRS, negative polarization near the interface blocks electron accumulation, leading to reduced electron trapping. This mechanism is consistent with the clockwise hysteresis observed in the FeSFET transfer characteristics (Figure 2e) and accounts for the distinct variation in subthreshold swing (SS) depending on the sweep direction. Figure 3h schematically illustrates the energy band diagrams of the FeSFET in the fatigue and recovery states, describing the behavior of electrons associated with interface trap density (*D*_it_) and *N*_ot_. In the fatigue state, degradation of the gate stack increases *N*_ot_, leading to enhanced trapping/de‐trapping activity and hindering OOP polarization switching under the gate field. After the CA process, the Joule heat effectively restores the *N*_ot_ within the gate stack and the previously discussed defects in α‐In_2_Se_3_ channel to near its initial level, thereby recovering the ferroelectric switching behavior. The recovery process successfully mimics the synaptic reuptake and vesicle refilling mechanisms observed in biological neural systems, as discussed earlier in Figure 1a.

**FIGURE 4:**
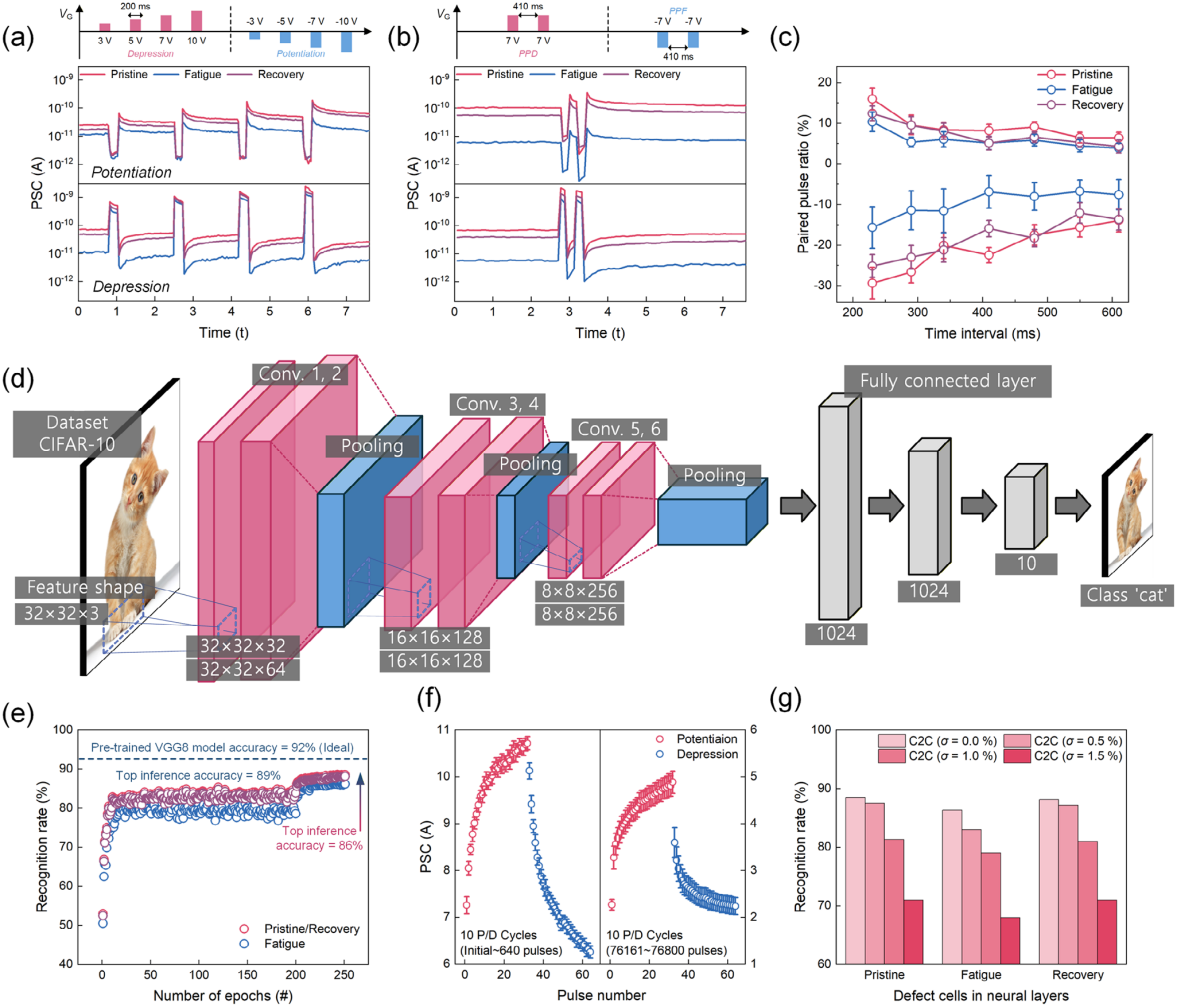
(a) The synaptic weight changes with different gate voltages, indicating STP characteristics. (b) The PPF and PPD effects with two consecutive pulses. (c)The PPF and PPD ratios are plotted with different pulse intervals. (d) Schematic of the VGG‐8 architecture based on the CIFAR‐10 dataset for evaluating a convolutional neural network (CNN). Image inference recognition rate when (e) pristine/recovery and fatigue state. (f) Mean and standard deviation of 10‐cycle potentiation/depression (left) pristine and (right) fatigue state, showing an increased standard deviation in the fatigue state. (g) Image recognition rate as a function of C2C variation.

The fatigue LTP discussed in Figure 2c indicates the degradation of long‐term memory and learning efficiency in the human brain caused by excessive external stimulation. Human memory and learning are regulated by dynamic changes such as rapid responses and information filtering, which are controlled through STP [26]. To investigate the synaptic characteristics of the FeSFET device depending on its state, electrical pulse responses under various stimulation conditions were evaluated, as shown in Figure 4a and Figure S14. The fatigue state shows an increase proportional to the pulse amplitude, pulse width, and read voltage, but exhibits lower conductance compared with the pristine state. When two pulses with different amplitudes and 200 ms widths are applied with an interval of 410 ms, paired‐pulse facilitation (PPF) and paired‐pulse depression (PPD) effects are observed, as shown in Figure 4b. Since PPF and PPD correspond to cumulative effects that occur before the post‐synaptic current (PSC) fully relaxes between pulses, the pulse interval must be adjusted appropriately. The paired‐pulse ratio (PPR) represents short‐term synaptic facilitation and is defined as *PPR*  =  (*A*_2_ − *A*_1_)/*A*_1_ × 100, where *A*_1_is the PSC under the first excitation and *A*_2_is the PSC under the second excitation. A larger PPR indicates that the residual effect of the first stimulus has a stronger influence on the second. The results obtained from seven devices are shown in Figure 4c. As the time interval decreases, the PPR becomes stronger, and the PPD induced by positive voltage shows a wider distribution than the PPF induced by negative voltage. This occurs because the higher trapped‐electron density in the gate oxide under the fatigue state disrupts synaptic facilitation. However, after the CA process is applied, the PPR in the recovered state returns to the same level as in the pristine state.

The results presented in this study are limited to the characteristics of a single FeSFET device. To provide a more comprehensive and practically applicable perspective, we further simulated the effect of CA at the array level, where the collective behavior of FeSFET synapses is reflected. In particular, compute‐in‐memory (CIM) architectures employ highly integrated crossbar arrays to enable ultra‐low‐power computational acceleration. These arrays consist of synaptic devices that offer fast switching, low operating voltage, and PSC. Thus, vector‐matrix multiplication (VMM) computations allow these arrays to realize artificial neural network (ANN) based applications.

The practical inference and training performance of neural networks is strongly influenced by several nonideal factors, including programming variation, NL, and stuck‐at‐fault defects [52, 53]. Accordingly, it is essential to evaluate neural network behavior at the array level while accurately reflecting the intrinsic characteristics of FeSFET. This study employs the NeuroSimV2.4 simulator, which incorporates device‐level nonidealities such as programming resolution, NL, and cycle‐to‐cycle (C2C) variation, thereby enabling quantitative assessment of the realistic hardware impact [54]. Using this platform, we performed VGG‐8 inference and training simulations on the Canadian Institute for Advanced Research (CIFAR‐10) dataset to quantitatively analyze the detrimental effects of FeSFET fatigue as well as the restorative impact of the CA process. Figure 4d illustrates the layers that constitute the VGG‐8 architecture, which serves as a reference for the subsequent discussion on defect cells [54, 55, 56]. A comparative table summarizing the key synaptic performance metrics relevant to system‐level evaluation of α‐In_2_Se_3_ FeSFETs, such as the number of states, operating current, endurance, and linearity, is provided in Table S2. The table includes not only newly designed α‐In_2_Se_3_‐based devices developed for functional enhancement, but also HfO_2_‐based FeFETs, two‐dimensional materials enabled by energy‐band engineering, organic materials, and Si‐based gate oxides. The performance metrics obtained in this study were optimized to enable a direct comparison of the limitations associated with the fatigue state and the effectiveness of the CA process relative to ideal recognition accuracy.

The simulation results incorporating the LTPS characteristics for CIFAR‐10 image classification are presented in Figure 4e. After training for up to 250 epochs, the validation accuracy reaches 89% in the pristine state, decreases to 86% in the fatigue state, and is restored to 88% after applying the CA process. Figure 4f presents the averaged potentiation/depression characteristics and corresponding standard deviations over 10 cycles for the pristine and fatigue states. In the pristine state, the NL values are 6.2 ± 0.11 for potentiation and 7.1 ± 0.18 for depression, whereas in the fatigued state, they increase to 29.2 ± 2.54 and 23.7 ± 2.91, respectively. The fatigue FeSFET exhibits a pronounced increase in standard deviation, indicating enhanced device nonuniformity arising from the increased *N*_ot_ and weakened polarization switching as the FeSFET enters the fatigue state. Consequently, these effects increase NL, preventing ideal synaptic‐conductance updates and leading to errors that reduce inference accuracy, ultimately degrading overall neural‐network performance. Furthermore, Figure 4g illustrates the relationship between recognition accuracy and NL under different C2C variation levels. C2C variation represents the current fluctuation instability in hardware inference systems, and device degradation during long‐term operation further amplifies this instability. The influence of C2C variation was observed to reach as high as 1.5%, with validation accuracy ranging from a maximum of 88.5% to a minimum of 68%.

**FIGURE 5:**
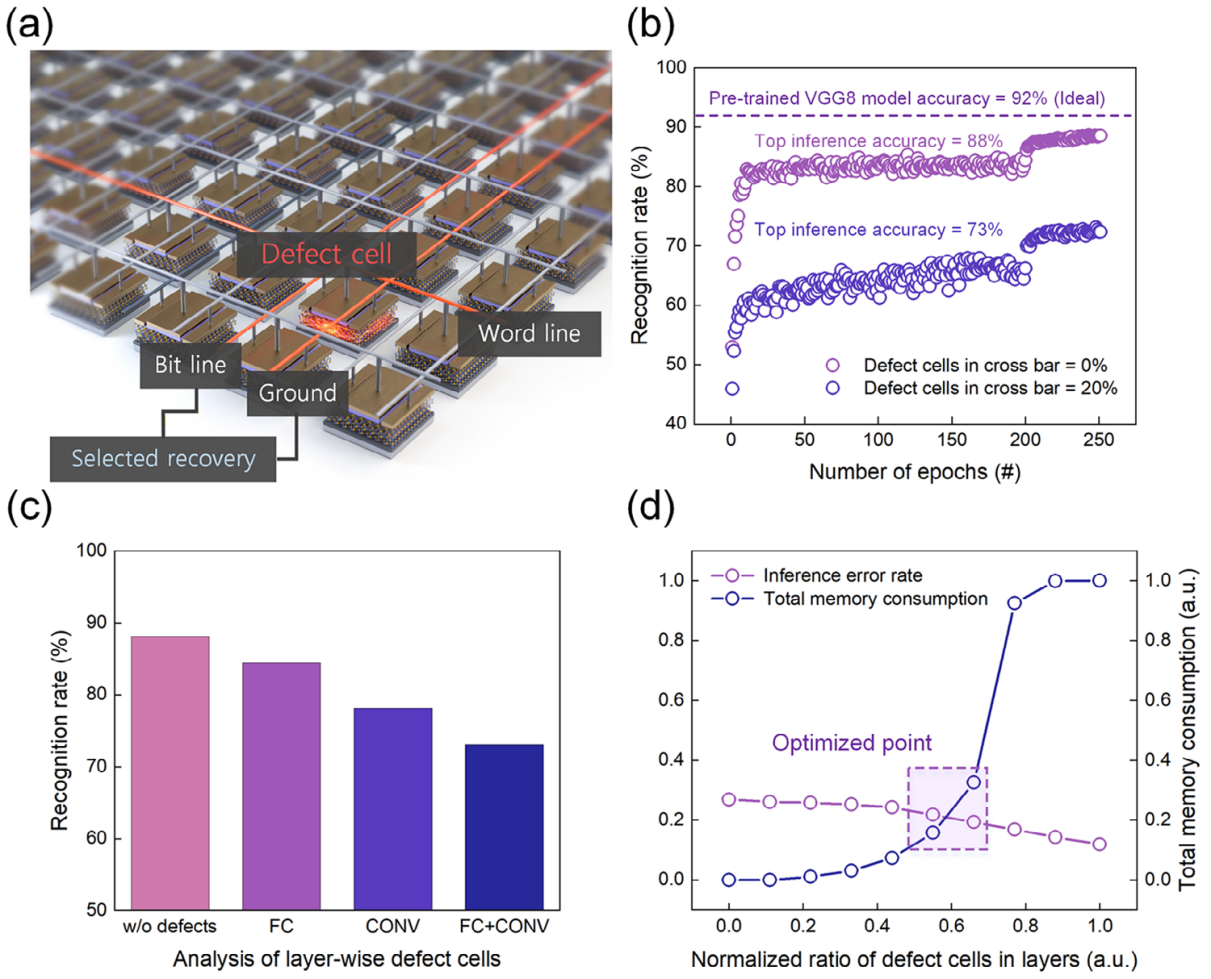
(a) Conceptual schematic of selective and localized recovery of arbitrary defect cells in a crossbar array through targeted annealing using the bit line, word line, and ground. (b) Recognition accuracy of the crossbar array under ideal conditions (without defect cells) and with randomly distributed defect cells. (c) Recognition accuracy of the VGG‐8 architecture when defect cells are introduced into specific layers. (d) Optimal process point for the CA process estimated from the trade‐off between inference error rate and total memory consumption.

In large‐scale computing systems, integrated devices may enter a fatigue state due to excessive operations, and as shown in Figure 5a, unexpected defect cells can appear owing to cell‐to‐cell interference. The CA technique proposed in Figure 2g involves applying a strong electric field between the bit line (drain) and ground (source) while accumulating charge through the word line (gate) to induce a transient high current that generates Joule heat. The selection scheme of the bit line and word line highlights that defect cells within a large‐scale array can be detected and locally annealed, enabling real‐time operation without external processing or delay during on‐site inference.

We further analyzed the degradation of neural network performance caused by stuck‐at‐fault behavior. A stuck‐at‐fault refers to a condition in which a synaptic conductance weight in the array becomes fixed due to fabrication‐related defects, preventing it from being updated during programming [57]. Such behavior inhibits accurate representation of synaptic weights within mathematical neural‐network models and ultimately leads to reduced inference performance. In particular, a stuck‐at‐LRS fault can cause excessive current flow. When unexpected defect cells arise due to FeSFET fatigue or cell‐to‐cell interference, the synaptic conductance may become so fixed that the device no longer exhibits measurable hysteresis, an outcome that severely degrades neural‐network performance. Therefore, compensation algorithms at the array level are essential to mitigate this issue.

This problem can be addressed by storing the locations of defect cells exhibiting stuck‐at‐fault behavior and incorporating this information into the backpropagation process through compensation algorithms [58, 59]. Figure 5b compares the recognition accuracy per epoch of the VGG‐8 network implemented with the FeSFET characteristics between the ideal case without stuck‐at‐fault defect cells and the case with 20% defective cells, revealing a performance gap of up to 15%. Figure 5c presents the recognition accuracy under the assumption that defect cells are located in specific layers of the VGG‐8 architecture. The VGG‐8 network comprises multiple convolutional and fully connected layers. Because image feature extraction is performed in the convolutional layers, defect cells located in these layers have a far more detrimental impact on recognition performance than those residing in the fully connected layers responsible for classification or regression tasks [58, 59].

While the results shown in Figure 5b,c highlight the necessity of annealing, the proposed CA process, although enabling sustainable operation of FeSFET‐based array structures, may incur unnecessary energy consumption due to the high‐voltage bias required to induce large transient currents. In addition, storing the locations of defect cells requires access to external memory, which undermines the energy‐efficiency benefits of CIM architectures and their VMM operations. Therefore, instead of applying CA to all defect cells, a partial‐annealing strategy can be adopted to mitigate both excessive energy consumption and the overhead of external memory access [60, 61]. Figure 5d shows the inference error rate and total memory usage as a function of the fraction of additional memory allocated to defect cell recovery. The x‐axis represents the synaptic‐weight parameters that constitute the entire VGG‐8 network; increasing x‐axis corresponds to the introduction of defect cells beginning from the convolution layers through the remainder of the array. The two y‐axis show the error rate and the memory overhead required when applying for the CA. As x‐axis increases, the number of defect cells also increases; however, once CA is applied, the error rate gradually decreases. At the same time, the memory required to store the defect‐cell locations increases. Ultimately, the intersection region of these two curves represents the optimal operating point for applying CA, where the degradation of neural‐network performance is minimized while the external memory overhead associated with CA is also kept to a minimum.

## 3 Conclusions

The CA process technique proposed in this study enables sustainable artificial synaptic mimicking using FeSFETs based on the two‐dimensional ferroelectric semiconductor α‐In_2_Se_3_. The synaptic depression process that occurs in biological neural networks under continuous stimulation, ferroelectric‐channel‐based neuromorphic systems also experience a fatigue state, in which the electrical conductivity gradually deteriorates. Simulations based on the CIFAR‐10 dataset reveal that as the device enters the fatigue state, the increasing NL and the location of defect cells within different architectural layers critically degrade image recognition accuracy. The CA process can perform localized annealing targeting specific defect cells in array structures; moreover, to prevent unnecessary power consumption, the optimal operating point for CA application is determined by evaluating the inference error rate and total memory usage. Because the CA process exploits the OOP and IP polarizations induced by the intrinsic asymmetric molecular structure of α‐In_2_Se_3_, it can effectively modulate electrical conductivity through high‐current‐induced Joule heat, making it highly compatible with FeSFET devices. The electrical pristine, fatigue, and recovery mechanisms of the FeSFET were elucidated using LFN spectroscopy, providing valuable insights into the precise analysis of LFN characteristics and the improvement of device reliability. Ultimately, the CA process, which mimics the reuptake and refilling processes observed in biological neural systems, can fundamentally mitigate synaptic fatigue, establishing it as a key enabler for realizing semi‐permanent ferroelectric‐channel‐based neuromorphic systems.

## 4 Experimental Section/Method

### 4.1 Device Fabrication

All devices were fabricated on highly doped p‐type silicon wafers with a 100 nm thermally grown SiO_2_ layer. α‐In_2_Se_3_ (2H, 2D Semiconductors) flakes were mechanically exfoliated using adhesive tape (224SPV, Nitto) and transferred onto the substrate via a dry‐transfer method. A photoresist (PR) layer was spin‐coated at 3000 rpm and soft‐baked on a 110°C hot plate for 90 s. The source and drain contact regions were defined using maskless lithography, followed by development to expose the contact areas of the α‐In_2_Se_3_ flakes. Subsequently, Ti/Au (5/70 nm) source and drain electrodes were deposited by electron‐beam evaporation. Information.

### 4.2 Material Characterization

The flakes were analyzed before and after doping using the same equipment and conditions. Photoluminescence (PL) and Raman spectrometer (FEX, WEVE) measurements were performed using to record PL and Raman spectra. Excitation was induced by 532 nm laser diodes. The thicknesses of the α‐In_2_Se_3_ flakes were determined using atomic force microscopy (AFM 5000, HITACHI) in Figure S1b. The THEMOS and PHEMOS images were obtained using the emission microscopy (EMMI) equipment (THEMOS‐Mini, PHEMOS‐2000/Hamamatsu) owned by QRT. An XPS (K‐Alpha+; ThermoFisher Scientific) was used to evaluate the chemical bond transitions in pristine, fatigue, and recovery states.

### 4.3 Device Characterization

The electrical characteristics of the FeSFET and CA process were evaluated at room temperature under ambient conditions using a semiconductor parameter analyzer (B1500A, Keysight Inc.), and synaptic behaviors with a waveform generator/high‐speed measurement device (B1530A, WGFMU, Keysight Inc.).

## Conflicts of Interest

The authors declare no conflicts of interest.

## Supporting information




**Supporting File**: advs75511‐sup‐0001‐SuppMat.docx.

## Data Availability

The data that support the findings of this study are available in the supplementary material of this article.
