# Supplementary material for: Sustainable Synaptic Device with Two‐Dimensional Ferroelectric Materials for Neuromorphic Computing
Source: Adv Sci (Weinh). 2026 May 7;13(40):e00064. doi: 10.1002/advs.202600064 (PMC13335525; doi:10.1002/advs.202600064)
Supplement: Supplementary file 1 — Supporting File: advs75511‐sup‐0001‐SuppMat.docx. [file ADVS-13-e00064-s001.docx]

Supporting Information

Sustainable Synaptic Device with Two-Dimensional Ferroelectric Materials for Neuromorphic Computing

Jaewook Yoo†, Seokjin Oh†, Minah Park, Jong Min Song, Hongseung Lee, Seohyeon Park, Sojin Jung, Seongbin Lim, Soohyun Lim, Dongsun Shin, Ji Won Heo, TaeWan Kim^*^, and Hagyoul Bae^*^

J. Yoo, M. Park, H. Lee, S. Park, S. Jung, S. Lim, S. Lim, D. Shin, H. Bae

Division of Electronic Engineering

Jeonbuk National University

Jeonju 54896, Republic of Korea

E-mail: <hagyoul.bae@jbnu.ac.kr>

J. M. Song, J. W. Heo, T. Kim

School of Advanced Fusion Studies and AI Semiconductor

University of Seoul

Seoul 02504, Republic of Korea

2D Epi, inc

567 Baekje-daero, Jeonju 54896, Republic of Korea

E-mail: <twkim@uos.ac.kr>

S. Oh, H. Bae

HB Inc.

567 Baekje-daero, Jeonju 54896, Republic of Korea

Keywords: 2D ferroelectrics, α-In_2_Se_3_, sustainable synaptic devices, current annealing (CA), convolution neural networks (CNN)


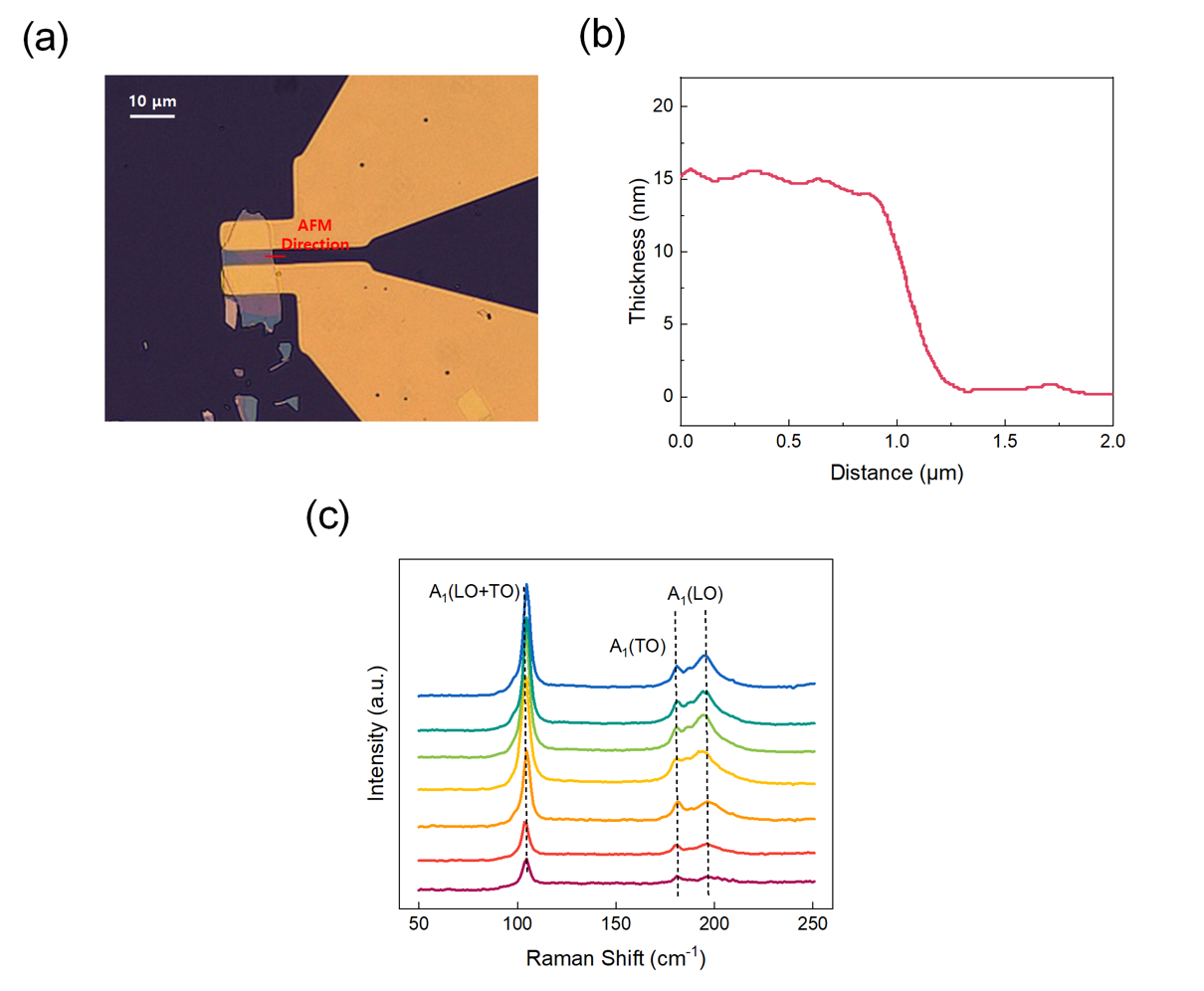


**Figure S1.** (a) Optical microscope image of the α-In_2_Se_3_ FeSFET. (b) Red line marked atomic force microscope (AFM) measurement area 15 nm thick α-In_2_Se_3_ channel measured by AFM. (c) Raman shift spectra indicating seven α-In_2_Se_3_ FeSFET.


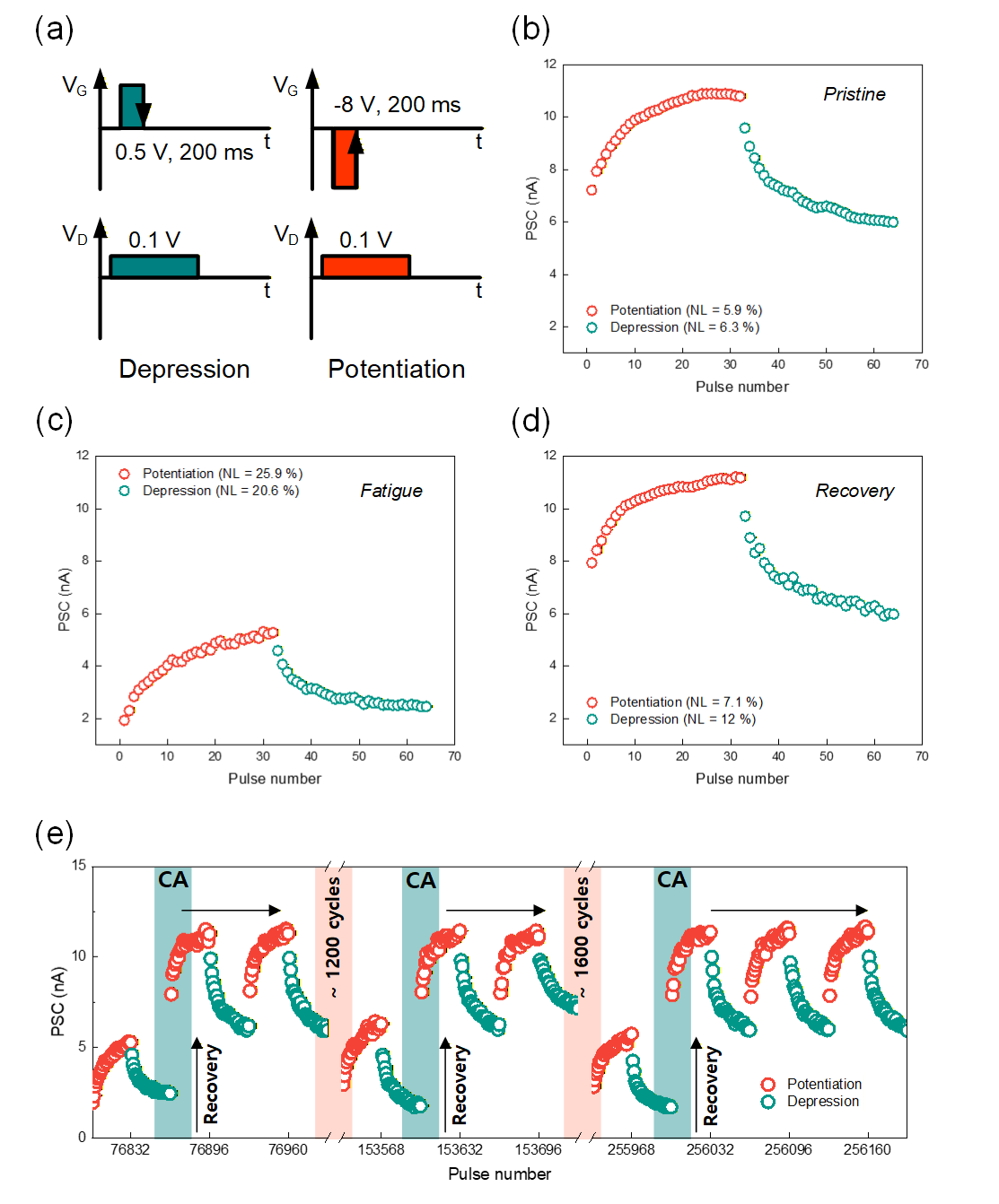


**Figure S2.** (a) Pulse measurement conditions applied for long-term potentiation and depression characteristics. 5-bit potentiation/depression behavior and non-linearity of the FeSFET (b) pristine, (c) fatigue, and (d) recovery state. (e) Degraded potentiation/depression (PD) curves after 1,200 and 1,600 cycles were restored through the second and third CA processes, respectively, demonstrating that the CA effect is repeatable rather than a one-time recovery phenomenon.


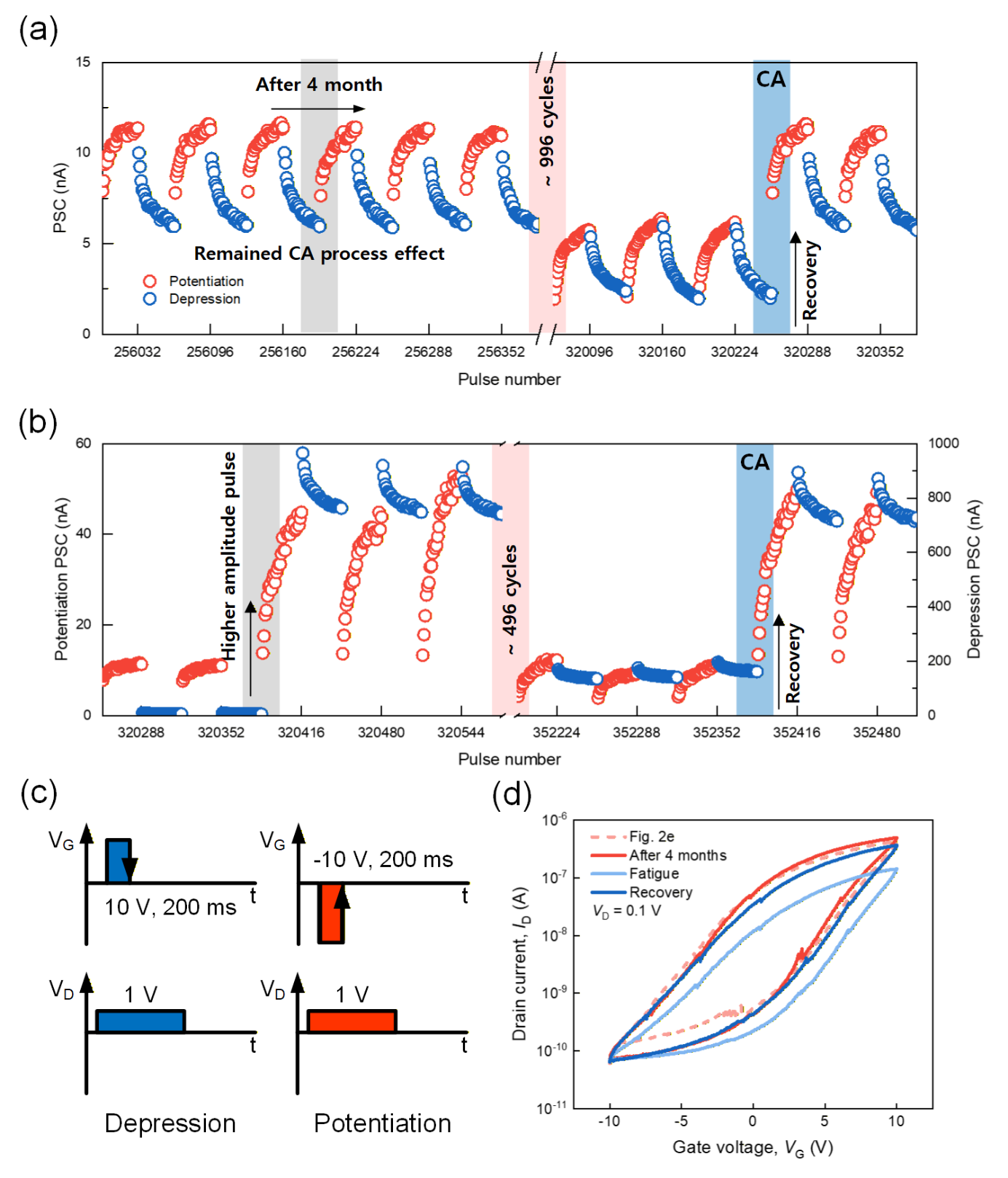


**Figure S3.** (a) LTP/LTD characteristics measured after four months to examine the persistence of the CA process effect, showing continued fatigue and recovery over 1000 cycles. (b) LTP/LTD characteristics obtained under higher-amplitude pulse over 500 cycles, exhibiting stronger degradation but successful recovery via the CA process. (c) Pulse measurement conditions used to obtain in Figure S3b. (d) Transfer characteristics overlaid for the recovered state in Figure 2e, the state after four months, and the fatigue and recovery states induced under the pulse conditions shown in Figure S3c.


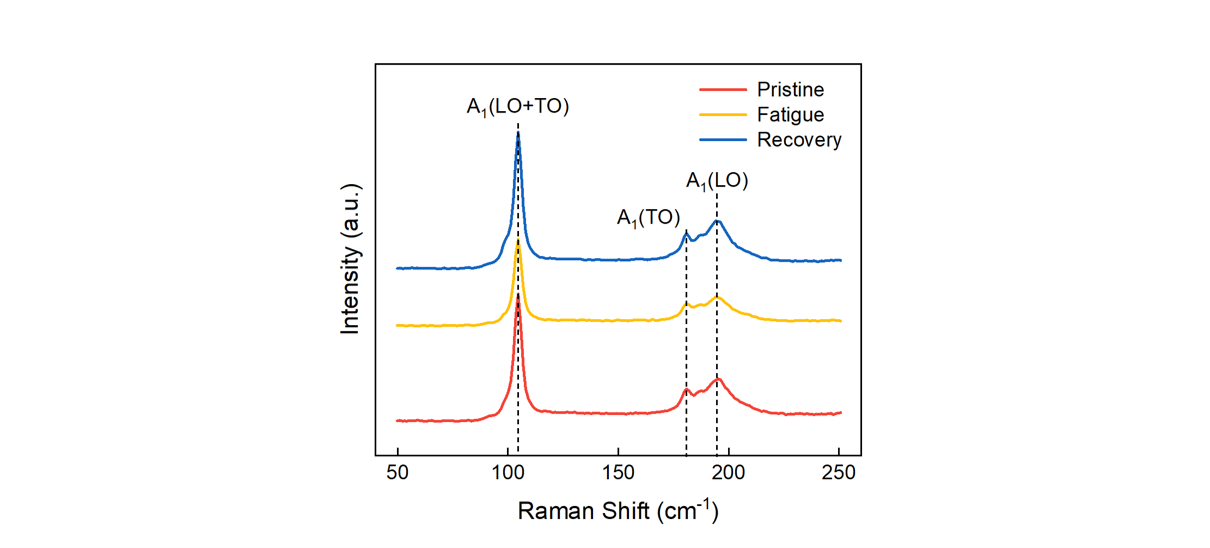


**Figure S4.** Raman shift spectra of the α-In_2_Se_3_ FeSFET measured in the pristine, fatigue, and recovery state.


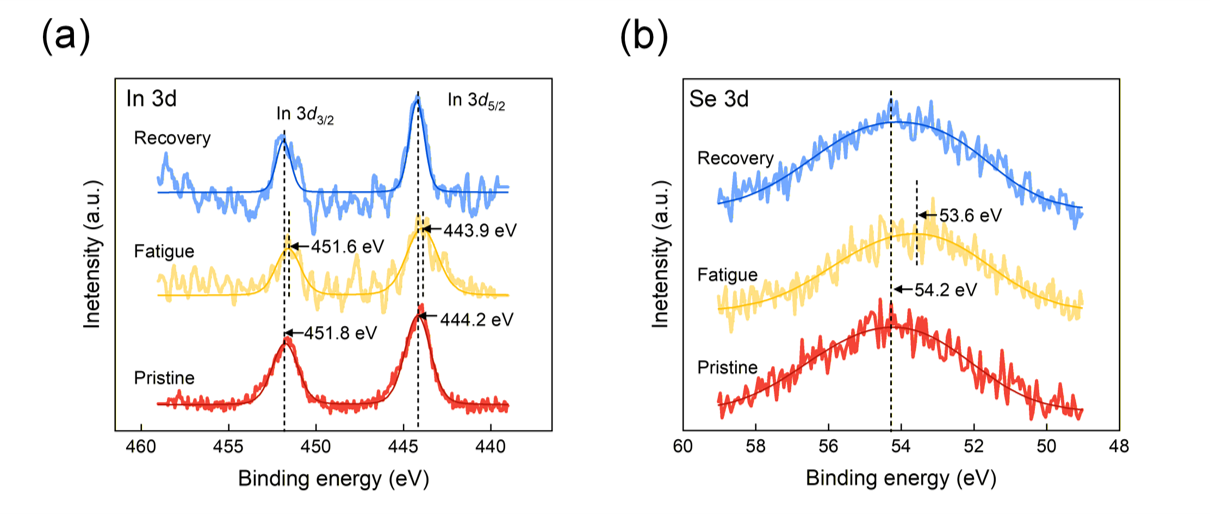


**Figure S5.** (a) In 3d, (b) Se 3d XPS spectra of the pristine, fatigue, and recovery state in α-In_2_Se_3_ FeSFET.


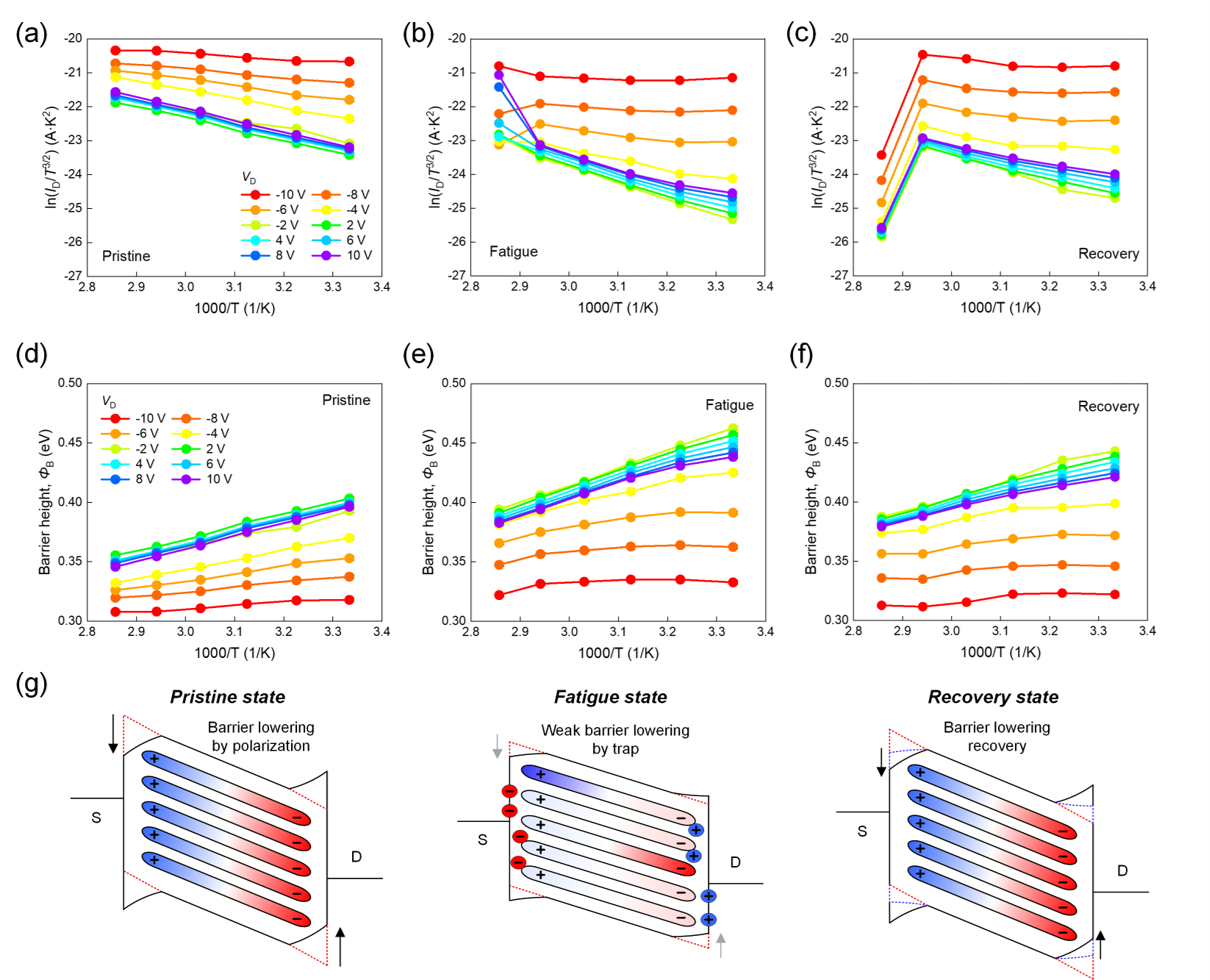


**Figure S6.** Arrhenius plot compared to the temperature of (a) pristine, (b) fatigue, and (c) recovery state in α-In_2_Se_3_ FeSFET. Schottky barrier height versus temperature of (d) pristine, (e) fatigue, and (f) recovery state in α-In_2_Se_3_ FeSFET. (g) Source to drain energy-band diagram of FeSFET states.

Arrhenius plot analysis, which can be employed to verify the thermionic emission model, follows the relation given by the following equation:

$I_{D}=AA^{*}T^{3/2}exp[-\frac{q}{k_{B}T}(\Phi_{B}-\frac{V_{D}}{\eta})]$ (1)

where A is the contact area, $A^{*}$ is the equivalent Richardson constant, *q* is the electron charge, $\Phi_{B}$ is the Schottky barrier height, $k_{B}$ is the Boltzmann constant, and ideality factor.


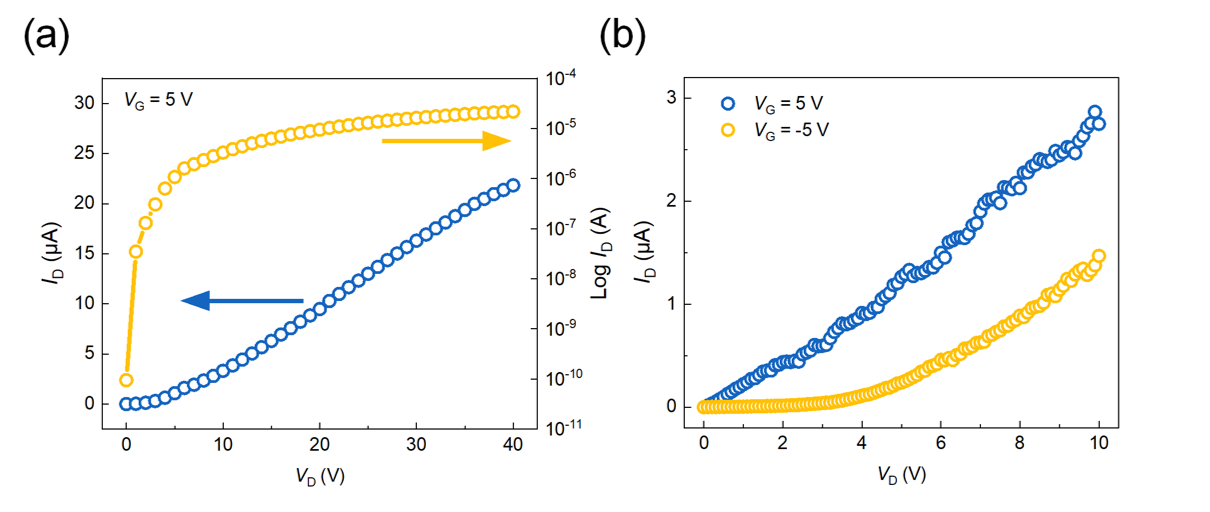


**Figure S7.** (a) Current flowing through α-In_2_Se_3_ FeSFET during Joule heat, showing ~20 µA under a pulse of *V*_pulse_ for 1 s in both linear and logarithmic output characteristics. (b) Output characteristics of the device show that positive and negative *V*_G_.


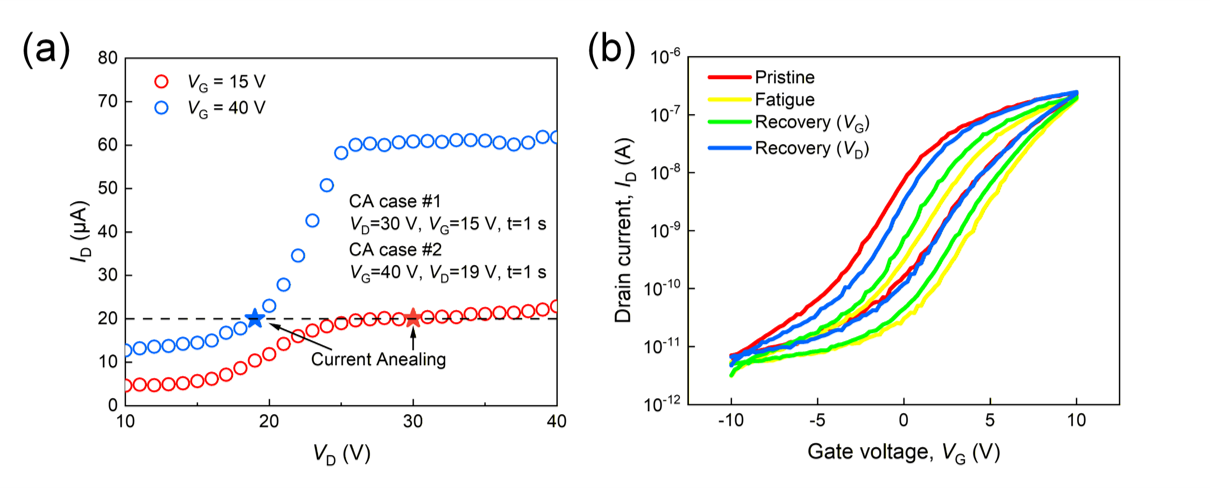


**Figure S8.** (a) Output characteristics of the FeSFET measured under applied gate voltages of 15 V, and 40 V. (b) Transfer characteristics of the FeSFET comparing the degree of recovery after applying the CA process under two different cases.


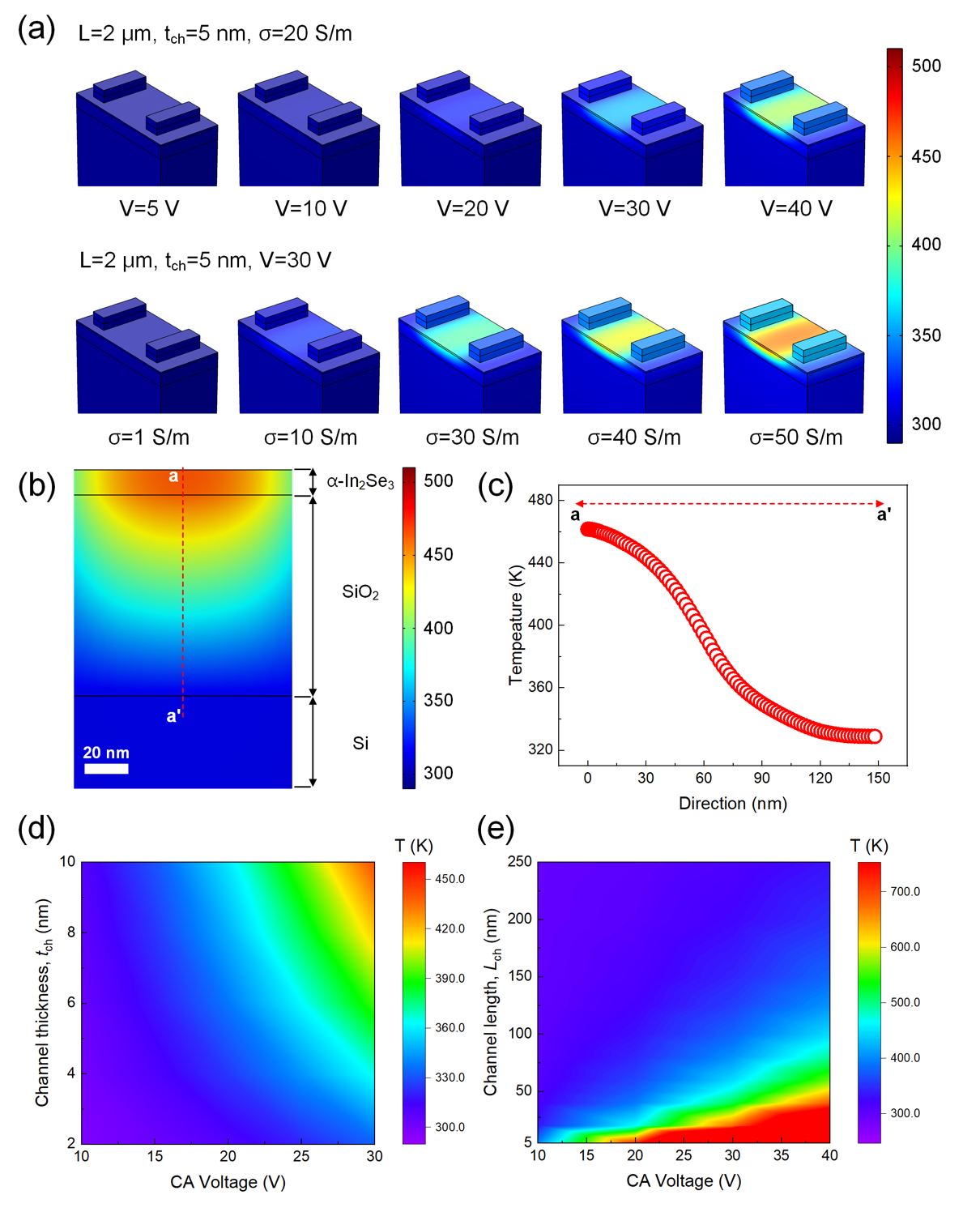


**Figure S9.** (a) Simulation data of the heat distribution profile in the α-In_2_Se_3_ FeSFET. Cross-section of the α-In_2_Se_3_ channel and SiO_2_ along a-a′ direction during the CA process, with corresponding (b) image and (c) temperature distribution. Simulated temperature according to (d) channel thickness (*t*_ch_) and (e) channel length (*L*_ch_) versus CA voltage.

|  | p-Si | SiO_2_ | α-In_2_Se_3_ | Ti | Au |
| --- | --- | --- | --- | --- | --- |
| Electrical conductivity  (S/m) | 1160 | 10^-9^ | [2] 1~50 | 3.1$\boldsymbol{\times}$10^6^ | 4.5$\boldsymbol{\times}$10^7^ |
| Thermal conductivity  (W/m∙K) | 100 | 1.4 | [3] 10 | 22 | 315 |
| Permittivity | 11.7 | 3.9 | [4] 17 | - | - |

**Table S1.** α-In_2_Se_3_ FeSFET composition material parameters applied in the COMSOL simulation.^[1]^


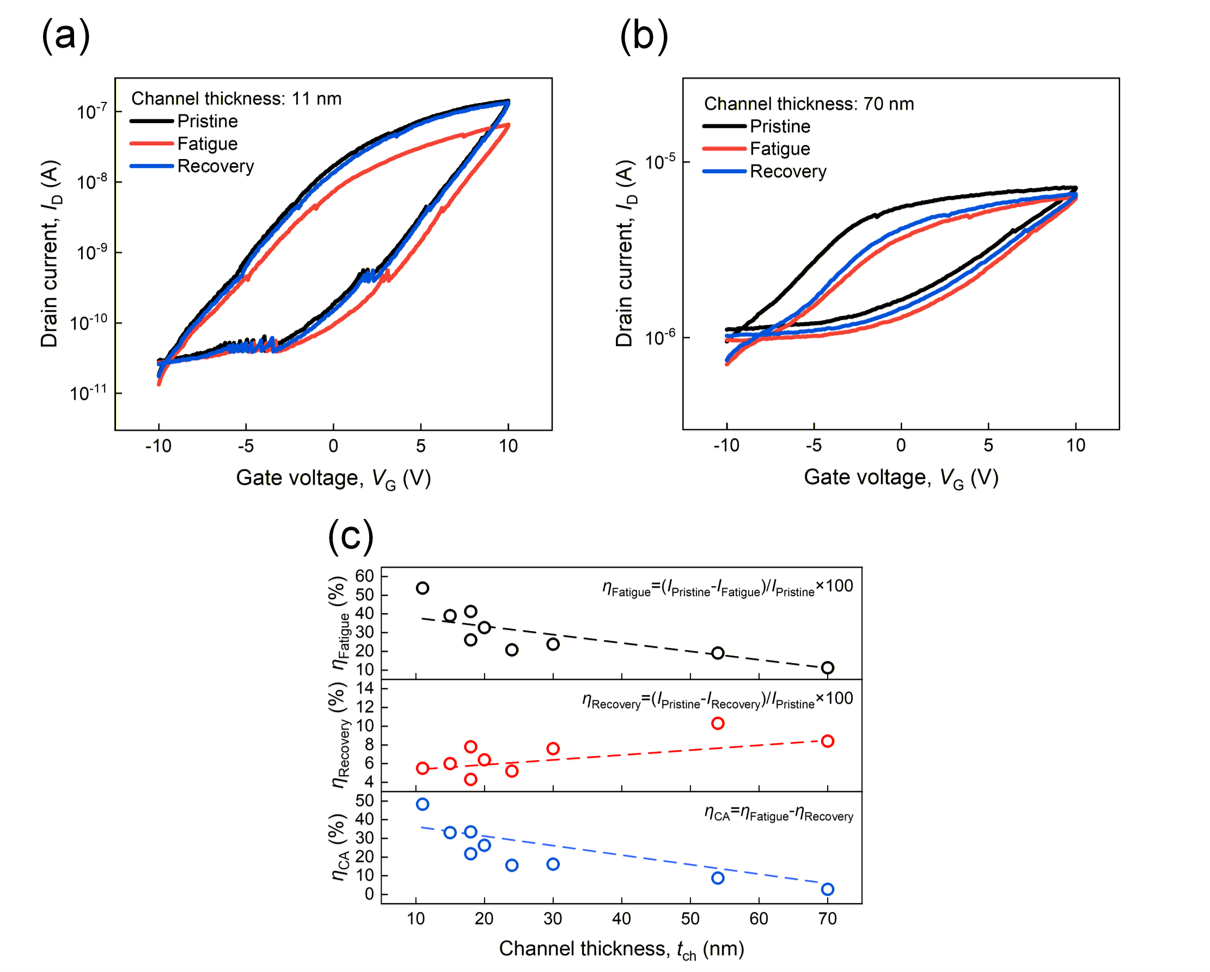


**Figure S10.** Transfer characteristics of the FeSFET with channel thickness of (a) 11 nm and (b) 70 nm, measured in the pristine, fatigue, and recovery state. (c) Fatigue efficiency, recovery efficiency, and CA efficiency as functions of *t*_ch_, with the corresponding calculation formulas included.


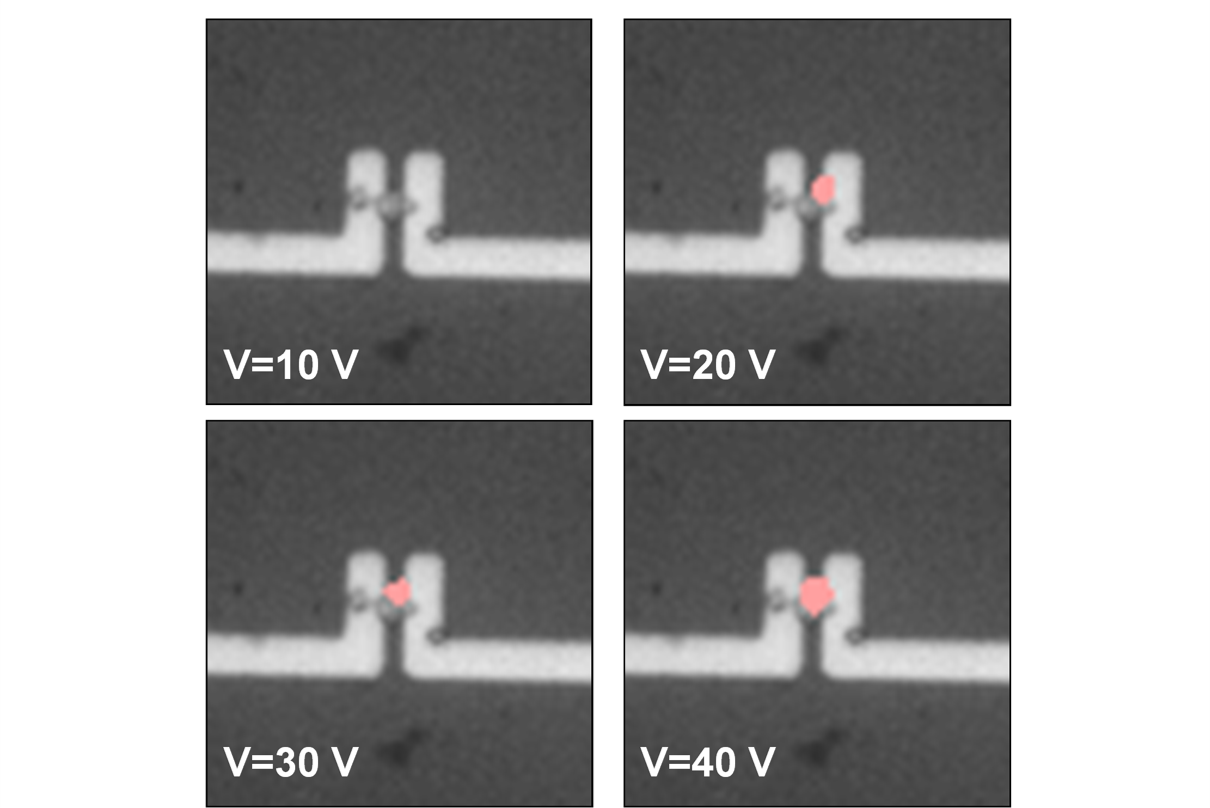


**Figure S11.** Voltage-dependent optical emission images obtained using the photon emission microscope (PHEMOS) system. THEMOS and PHEMOS images, typically used in emission microscopy (EMMI) analysis to locate defects such as opens, shorts, and leakages, were employed in this study to verify Joule heating and light emission.


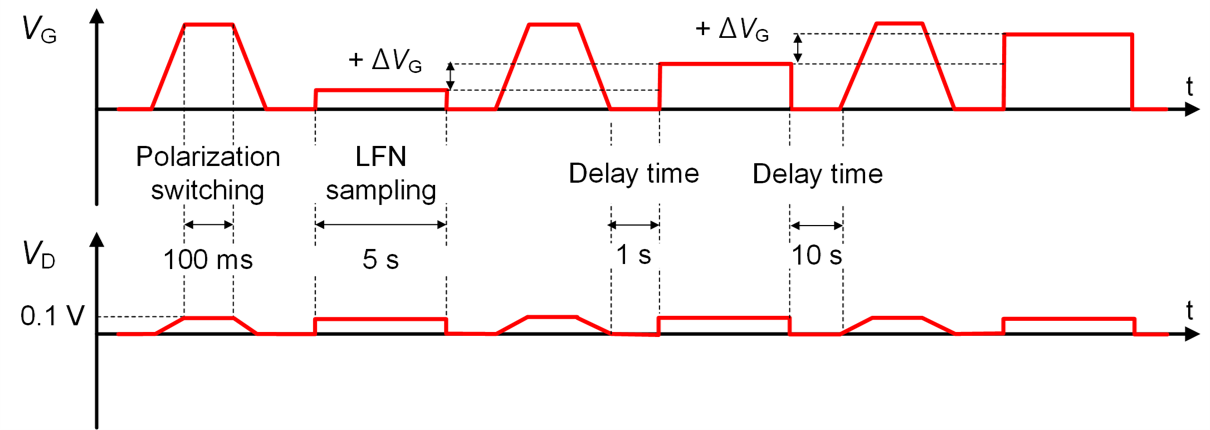


**Figure S12.** The low-frequency noise (LFN) measurement procedure is used to extract the noise characteristics of the FeSFET. Simultaneous *V*_G_ and *V*_D_ pulsing programs OOP/IP polarization switching and stabilize the *I*_D_. LFN sampling is performed after a 1s delay, with 10s intervals between measurements for stability.


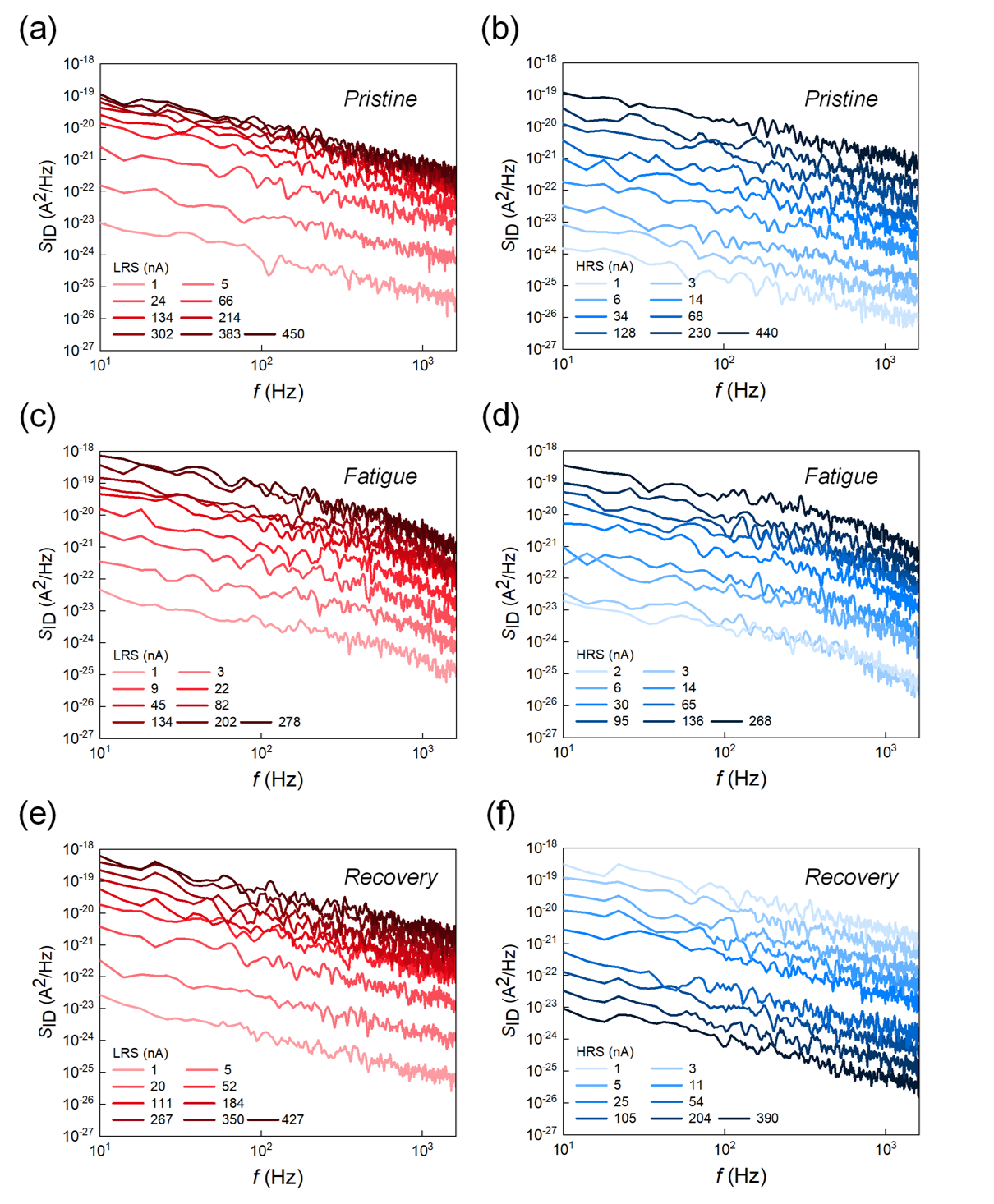


**Figure S13.** *S*_ID_ versus frequency for the α-In_2_Se_3_ FeSFET with pristine ((a) LRS, (b) HRS), fatigue ((c) LRS, (d) HRS), and recovery ((e) LRS, (f) HRS), respectively. The *V*_D_ is set at 0.1 V during the measurement.


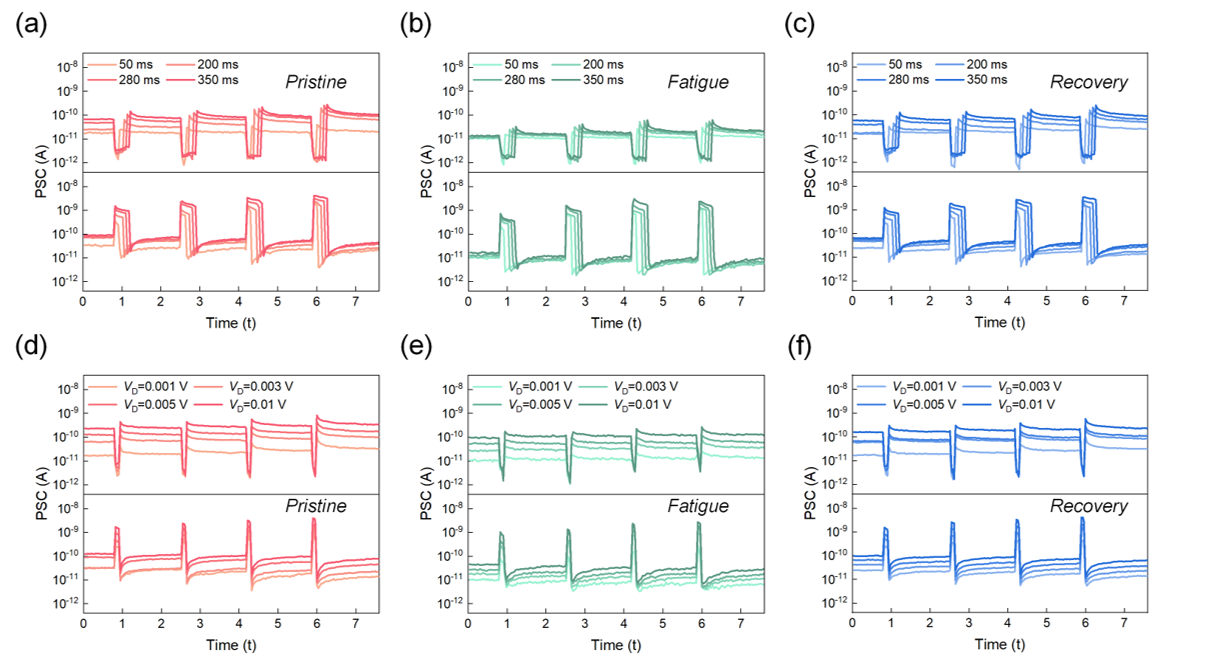


**Figure S14.** Synaptic weight modulation exhibiting STP characteristics. (a) Pristine, (b) fatigue, and (c) recoverey FeSFET driven by gate pulses with amplitudes of 3, 5, 7, and 10 V, where the pulse width is split and *V*_D_ is fixed at 0.001 V. Corresponding measurements for (d) pristine, (e) fatigue, and (f) recovery FeSFET under gate pulses with amplitudes of 3, 5, 7, and 10 V and a fixed pulse width of 50 ms, while *V*_D_ is split.

|  | Number of  states | Operating current | Weight update  power | Endurance | Switching speed | Linearity |
| --- | --- | --- | --- | --- | --- | --- |
| This work (α-In_2_Se_3_) | 64 | 1~10 nA | 200 pJ | > 3.5 **×** 10^5^ | 200 ms | 93~94 % |
| FeSFET (α-In_2_Se_3_/h-BN) ^[5]^ | 100 | 20~150 nA | 10 nJ | > 6 **×** 10^4^ | 100 ms | 70~83 % |
| FeSFET (α-In_2_Se_3_, PEA) ^[6]^ | 200 | 20 μA | 200 pJ | > 2 **×** 10^3^ | 10 ms | 95~97 % |
| FeSFET (α-In_2_Se_3_/Al_2_O_3_) ^[7]^ | 100 | 100~500 nA | 30 pJ | > 4 **×** 10^3^ | 100 μs | 83~90 % |
| FeSFET (α-In_2_Se_3_, IP anisotropy) ^[8]^ | 200 | 0.5~2 μA | 50 pJ | > 4 **×** 10^3^ | 100 μs | 93~98 % |
| 2-terminal device (Graphene  /α-In_2_Se_3_, Photoresponsivity) ^[9]^ | 128 | 3~25 nA | 0.4 mW/cm^-2^ | N/A | 40 ns | 94~97 % |
| FeSFET (α-In_2_Se_3_/h-BN, Photoresponsivity) ^[10]^ | 60 | 1 ~ 10 nA | > 1 mW/cm^-2^ | > 3 **×** 10^2^ | 0.001~5 s | 76~92 % |
| Ferroelectric junction  (α-In_2_Se_3/_SiO_2_) ^[11]^ | 200 | 100 μA | 5 μJ | > 1 **×** 10^2^ | 100 ms | 88~91 % |
| FeFET (HZO/Si) ^[12]^ | 32 | 0.3~10 μA | 400 pJ | > 1 **×** 10^5^ | 10 μs | 86~90 % |
| FeFET(HZO/Si) ^[13]^ | 100 | 30~180 μA | 10 pJ | > 1 **×** 10^4^ | 100 μs | 82~87 % |
| FeFET (HZO/IGZO) ^[14]^ | 200 | 1~10 nA | 20 pJ | > 1 **×** 10^4^ | 200 μs | 93~98 % |
| FeFET (HZO/WS_2_) ^[15]^ | 300 | 10 nA | 200 pJ | > 2 **×** 10^2^ | 10 ms | 72~74 % |
| FeFET (HZO/SnS_2_) ^[16]^ | 140 | 1~10 μA | 10 pJ | > 1.4 **×** 10^4^ | 1 μs | 87~88 % |
| FeFET (CWCNT/P(VDF-TrFE)) ^[17]^ | 120 | 1 ~ 9 nA | 100 nJ | > 5 **×** 10^2^ | 500 ms | 90~94 % |
| 3-T artificial heterosynpatic device (MoO_x_/MoS_2_) ^[18]^ | 60 | 1 nA | 0.1 fJ | > 1 **×** 10^3^ | 100 ns | 88~90 % |
| MISFET (MoS_2_/CuInP_2_S_6_/MoS_2_) ^[19]^ | 120 | 4 ~18 nA | 1 nJ | > 2 **×** 10^3^ | 0.2 s | 88~91 % |
| Charge trapping gate (BP/PO_x_) ^[20]^ | 40 | 2 μA | 400 nJ | N/A | 10 ms | 85~89 % |
| Floating gate (MoS_2_/h-BN) ^[21]^ | 60 | 100 nA | 100 pJ | > 3 **×** 10^2^ | 1 ms | 80~98 % |
| Heterostructure (ReS_2_/BP) ^[22]^ | 100 | 100 nA | 5 pJ | > 3 **×** 10^2^ | 10 μs | 82~85 % |
| Si (Gated Schottky diode) ^[23]^ | 30 | 0.1~10 mA | 100 nJ | N/A | 10 μs | 90 % |
| Si (Si/SiO_2_/Si_3_N_4_ gate stack) ^[24]^ | 60 | 1~10 μA | 100 pJ | > 1 **×** 10^5^ | 5 μs | 92~94 % |

**Table S2.** Comparison of α-In_2_Se_3_ based artificial synapse with typical reported synaptic transistors to the publish papaers. Our work systematically investigates the continuous imporvement of endurance through the CA process the resulting impact of linearity modulation at the system level.

**References**

1. J.-H. Cha, I. Lee, S. W. Yun, et al., *Nanoscale*, 2025, **17**, 11305.

2. Z. Shi, Y. Xiao, M. Li, et al., *Adv. Mater.* 2025, e07536.

3. S. Zhou, X. Tao, Y. Gu, *J. Phys. Chem. C* 2016, 120, 4753.

4. D. Wu, A. J. Pak, Y. Liu, et al., *Nano Lett.* 2015, **15**, 8136.

5. X. Zhang, J. Zhou, Y. Zhang, et al., *Adv. Sci.* 2025, **12**, 2502286.

6. J.-H. Kim, S.-H. Kim, H.-Y. Yu, *Small* 2024, **20**, 2405459.

7. L. Wang, X. Wang, Y. Zhang, et al., *Adv. Funct. Mater.* 2020, **30**, 2004609.

8. J.-H. Kim, S.-H. Kim, H.-K. Jin, et al., *Adv. Mater.* 2025, e17849.

9. J. Zeng, G. Feng, G. Wu, et al., *Adv. Funct. Mater.* 2024, **34**, 2313010.

10. S.-J. Kang, W. Jung, O. H. Gwon, et al., *Small* 2024, **20**, 2307346.

11. Y.-R. Jeon, D. Kim, C. Biswas, et al., *Adv. Mater.* 2025, **37**, 2413178.

12. W. Shin, J. Byeon, R.-H. Koo, et al., *Adv. Sci.* 2024, **11**, 2307196.

13. M. Seo, M.-H. Kang, S.-B. Jeon, et al., *IEEE Electron. Device Lett.* 39, 1445-1448.

14. D. Kim, H. Jeong, G. P. Pyo, et al., *Adv. Sci.* 2024, **11**, 2401250.

15. L. Chen, L. Wang, Y. Peng, et al., *Adv. Electron. Mater.* 2020, **6**, 2000057.

16. C.-M. Song, D. Kim, S. Lee, H.-J. Kwon, *Adv. Sci.* 2024, **11**, 2308588.

17. S. Jang, S. Jang, E.-H. Lee, et al., *ACS Appl. Mater. Interfaces* 2019, **11**, 1071-1080.

18. W. Huh, D. Lee, S. Jang, et al., *Adv. Mater.* 2023, **35**, 2211525.

19. J. Chen, Z. Wen, F. Yang, et al., *Nat. Commun* 2025, **16**, 702.

20. H. Tian, Q. Guo, Y. Xie, et al., *Adv. Mater.* 2016, **28**, 4991-4997.

21. C. He, J. Tang, D.-S. Shang, et al., *ACS Appl. Mater. Interfaces* 2020, **12**, 11945-11954.

22. X. Xiong, J. Kang, Q. Hu, et al., *Adv. Funct. Mater.* 2020, **30**, 1909645.

23. J.-H. Bae, S. Lim, B.-G. Park, J.-H. Lee, *IEEE Electron. Device Lett.* 2017, **38**, 1153-1156.

24. J. Hur, B. C. Jang, J. Park, et al., *Adv. Mater.* 2018, **28**, 1804844.
